# Complex network dynamics of the topological structure in a geochemical field from the Nanling area in South China

**DOI:** 10.1038/s41598-020-76905-6

**Published:** 2020-11-13

**Authors:** Nian Peng, Xiaoyan Zhu, Yongshun Liu, Baofeng Nie, Ying Cui, Qianqian Geng, Chongwen Yu

**Affiliations:** 1 College of Resource Environment and Tourism, Capital Normal University, 105 West Third Ring Road North, Haidian District, Beijing, 100048 China; 2 School of Earth Sciences and Resources, China University of Geosciences (Beijing), Beijing, 100083 China

**Keywords:** Environmental sciences, Solid Earth sciences

## Abstract

The topological classification of geochemical elements is widely used as a reference for regional prospecting prediction. In this study, we analyze the topological correlation structures of 39 representative geochemical elements from the Nanling area of South China by implementing the complex networks theory. The topological correlation structures of geochemical elements have a high clustering coefficient (0.8120–0.8880), but the magnitude of the shortest path (1.2950–2.3600) is small. In combination with the analysis of complex networks characteristics, we report that the topological correlation structures of the geochemical elements in this area have small-world characteristics, which reveals the self-organized criticality. As shown in the topological network, two random elements have some level of associations, which present a specific community feature. Our preliminary result shows that with changing the control parameter (*k*) of “coarse-graining”, the topological correlation structures undergo two critical phase transitions. As the control parameter (*k*) reaches 0.44, the entire element system evolves into two parts. When the control parameter (*k*) reaches 0.63, the system forms three “communities”. It is worth noting that the three “communities” are basically consistent with the Goldschmidt’s geochemical classification of the elements, which are lithophile, siderophile, and chalcophile groups, respectively. In these “communities”, we also found that a small level of component units is nested.

## Introduction

Complex networks are defined as large-scale systems with some or all the properties of self-organization, self-similarity, presence of attractors, small-world, and free-scale. They are characterized by nodes, edges, and topological matrices^1^. Due to the advancement in small-world effect^2^ and scale-free theory^3^ in the last 20 years, complex networks theory has been proactively exploited as a descriptive and empirical research tool for various network types, such as river systems^4,5^, food chain networks^6^, logistics networks^7,8^, financial markets^9^, natural resources^10^, etc.

Nowadays, the investigation of complex networks in many diverse fields has become the latest research hotspot and a challenging topic at the frontiers of science^11,12^. This method has a strong significance to the study of the specific interactive relationship of complex systems^11,13,14^.

The Nanling area of South China is an open complex giant system with abundant reserves of non-ferrous metal mineral resources. This area witnesses frequent magmatic activity. Chinese research groups have carried out numerous investigative studies on the mineral resources of the Nanling area in recent years^15–20^. These studies focused primarily on the mineralization potential, deposit types, metallogenic processes and characteristics, and prospecting prediction. Complexity science and nonlinear science theory are seldom applied in this area. Only Yu Chongwen^21–23^ used complexity theory in his works to explain the mineralization of the Nanling area. Yu Chongwen^24,25^ pointed out that the combination of mathematics, chemistry, physics, nonlinear science, and complexity theory with geological science facilitated a long-term exploration of the nature of geological phenomena and addressed the basic problems of geological sciences.

In the paper, we present a case study of geochemical elements from 1:200,000 stream sediment samples in the Nanling area in South China. We have constructed the complex network topological correlation structure charts of 39 main geochemical elements. Moreover, we also discuss the dynamic evolution process of the complex networks structure of the geochemical field using complexity science theory and reveal the symbiotic combination rule under the geochemical element topology in the area. This provides a practical reference for comprehensive prospecting and effective resource utilization.

## Materials and methods

### Regional overview and geochemical characteristics

#### Overview of the study area

The Nanling area is in the south-central part of South China. It has a total area of about 200,000 km^2^ that encompasses central-southern Hunan, northern Guangxi, southern Jiangxi, and northern Guangdong. There are abundant early Mesozoic granites and rift basins with mainly east–west and north-east distribution direction of the structural belt. The mountain system is arranged in nearly east–west, which is different from the basins and mountains in the south-east coastal area. The strata in this area are characterized as Devonian, followed by Cambrian, while as Sinian and lower Carboniferous in Hunan, Jiangxi, and Guangdong Province. The rock bodies comprise mostly the Yanshanian granite and subordinate Caledonian granite. This region has experienced multiple tectonic–magmatic-metallogenic events that resulted in several large and super-large mineral deposits, e.g., the middle Jurassic Baoshan porphyry Cu–Mo deposits, the late Jurassic Shizhuyuan skarn W–Sn deposit, and Huangshaping skarn Pb–Zn deposit. In addition to South China being the metallogenic prospect zone of the non-ferrous metals, rare earth elements (REE), and radioactive minerals^26^, this area is also rich in W, Sn, Nb, Ta, U, and other metal mineral resources, which has long been a focus of geological research.

#### Regional geochemical characteristics

**Figure 1:**
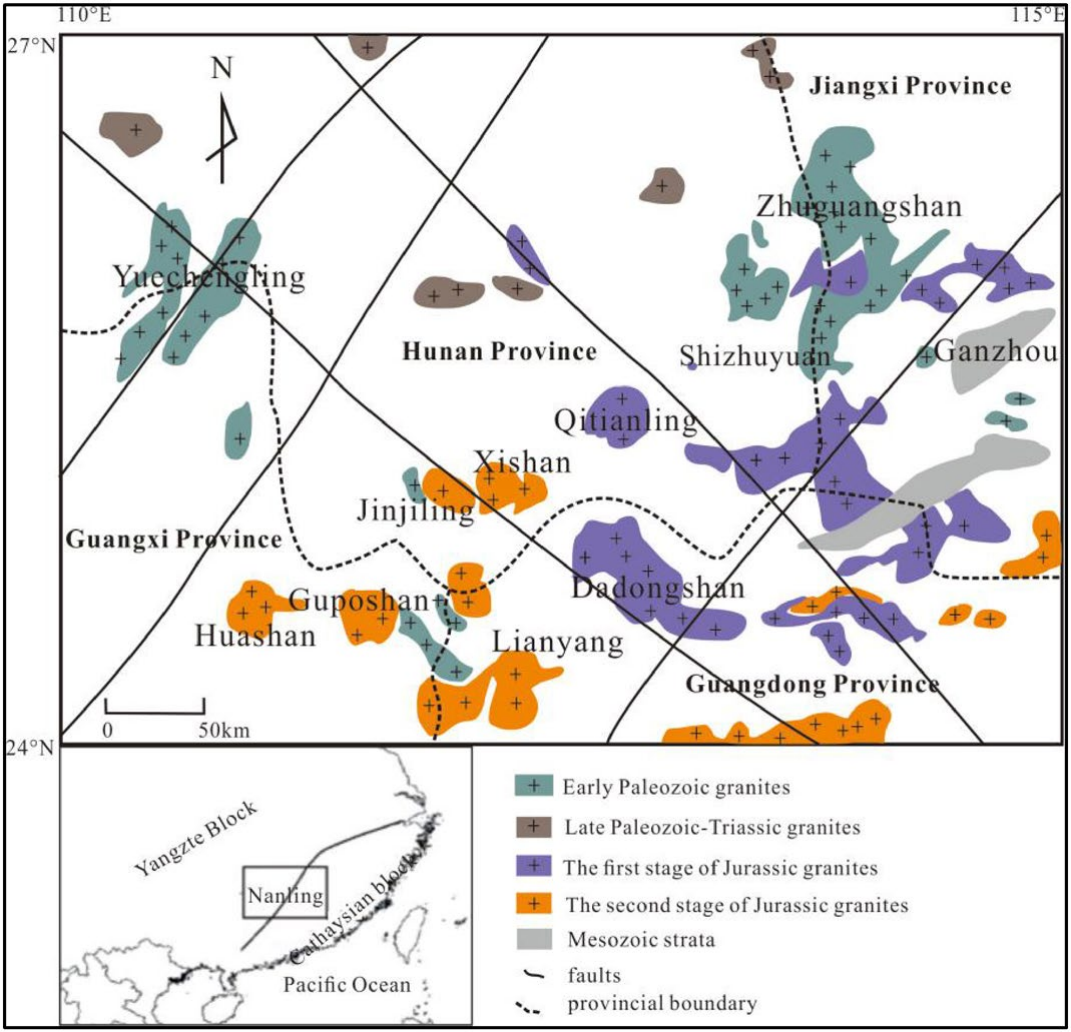
Geological and mineral resources map of the Nanling area. This figure was created with ArcGIS 10.5 (https://www.esri.com/en-us/arcgis/products/index).

The Nangling area in South China is located at the juncture between the Yangtze Block and the Cathaysian block (Fig. 1). This region possesses many enrichment layers and ample deposits of W, Sn, Sb, As, U, Pb, Zn, and REE^27^. The high geochemical backgrounds of W, Sn, and Bi content are mainly distributed in the central and eastern parts of the Nanling area, showing a distinct northeastward belt distribution, which is consistent with its essential geotectonic belt distribution. The high content in the geochemical background shows the potential resource advantages of these elements in this region. The geochemical backgrounds of Pb and Zn are quite different. Pb is primarily distributed on the border area between Hunan and Guangdong Province. In contrast, Zn is mainly distributed in the middle and west of the Nanling area. Au and Ag have an uneven geochemical distribution with a high degree of dispersion^28^. Besides, the main ore-forming elements in the Nanling area have distinct geographical locations. For example, the elements including W, Sn, Pb, Zn, Mo, and Ag are the main ore-forming elements in the southern Hunan Province; the elements Sn, Pb, Zn, and Sb are the main ore-forming ones in the northern Guangxi Province; Sn, Pb, Zn, Ag, and W are the main ore-forming ones in the northern Guangdong Province; and W and Sn are the main ore-forming ones in the southern Jiangxi Province. The Nanling area has become one of the premier choice areas to solve the shortage of domestic mineral resources^29^.

### Data sources

The primary research data used in this article is derived from a sub-topic of the Geological and Mineral Investigation and Evaluation Project of the key metallogenic belt of the China Geological Survey (1212011121101). It is the original geochemical data used by the Nanling working group for the mineral resource evaluation of the China Geological Survey in preparation for the Geophysical and Geochemical Exploration Remote Sensing Information Atlas (https://geocloud.cgs.gov.cn/). Based on the 1:200,000 stream sediment geochemical exploration of Hunan, Guangxi, Jiangxi, and Guangdong provinces with 43,584 sampling points in total, our research involves 39 representative elements, including Ag, As, Au, B, Ba, Be, Bi, Cd, Co, Cr, Cu, F, Hg, La, Li, Mn, Mo, Nb, Ni, P, Pb, Sb, Sn, Sr, Th, Ti, U, V, W, Y, Zn, Zr and Al_2_O_3_, CaO, Fe_2_O_3_, K_2_O, MgO, Na_2_O, and SiO_2_, with a total of 1,656,230 data. After preprocessing the data and eliminating some problematic sampling points, 40,029 valid sampling points are selected. The total valid data is 1,561,131. Data collected were processed and calculated using IBM SPSS Statistics, while the Gephi Social Network Analysis software was used for visualization.

### Theory and methodology

#### Topological characteristics based on complex networks

The complex network has small-world and scale-free properties. However, with continuing research, the community structure has become an essential new feature that reveals the complex structure of the network. Generally, the connection between nodes is relatively tight within the same community, while the connection between nodes is relatively sparse between different communities. The research on the network community structure mainly uses various methods of community detection to mine the topology structure of the complex networks and analyze the characteristics of network community structure, to better understand its structure and function.

The topological structure is one of the main contents of our research on complex networks theory. A network can be classified according to the topological characteristics of complex network. A complex network is composed of nodes, connected edges, and a topological matrix. It can be regarded as a set of points and lines, which can be expressed as $G = (V,E)$, wherein, $V$ is a set of all nodes in the network; $E$ is a set of all connected edges between pairs of nodes in the network. If the connected edges corresponding to node pair $(i,j)$ and node pair $(j,i)$ are the same edge, then this network is called an undirected network; otherwise, it is a directed network. If all the connected edges in the network represent equal values, the network is an unauthorized network; else, it is a weighted network^30^.

#### Pearson correlation coefficient

The Pearson correlation coefficient is a statistical index that reflects the strength of the relationship between two variables. The advantage of calculating the Pearson correlation multiple times is that it corrects the system error by measuring the “distance” between one element and the other elements of the whole system. Its specific application steps are as follows: (1) calculate the value of the correlation coefficient of each element for sample data as the initial coefficient matrix (*X*_0_); (2) continue to calculate the correlation coefficients among the columns of the matrix (*X*_0_), and obtain the “correlation coefficient of the correlation coefficient” and a new matrix (*X*_1_); (3) continue to carry out the steps above, and calculate the correlation coefficient between the columns, and obtain another new matrix (*X*_2_) and (4) the final matrix is formed by the “correlation coefficient of the correlation coefficient” after recursively repeating the above steps. The iterative process is shown in Eq. (1) as follows:

1 $$\begin{gathered} X_{i+1} = R(X_{i}) \hfill \\ X_{0} = R(M) \hfill \\ \end{gathered}$$

where *M* denotes a matrix consisting of 39 elements as columns and 40,029 sample spots of each element as rows (Supplementary Appendix Table I). *R* is the Pearson correlation, and *X*_0_ is an initial 39 × 39 coefficient matrix. The formula (2) showing the calculation of the correlation coefficient (*R*) is as follows:

2 $$R_{ab} = \frac{{\sum\nolimits_{i = 1}^{N} {(a_{i} - \overline{a} )(b_{i} - \overline{b} )} }}{{\sqrt {\sum\nolimits_{i = 1}^{N} {(a_{i} - \overline{a} )^{2} } } \sqrt {\sum\nolimits_{i = 1}^{N} {(b_{i} - \overline{b} )^{2} } } }}$$

where in *N* represents the number of sample points, *a* and *b* represent the elements. Additionally, *a*_*i*_ and *b*_*i*_ represent the content of the element *a* and *b* at the *i*th sampling point, respectively. Through the formula mentioned in Eq. (2), we can get the correlation coefficient *X*_0_ of the element content matrix *A*_39 * 40029_ in the Nanling area and then obtain the stronger correlation coefficient matrix *X*_1_ by recalculating the Pearson correlation coefficient.

Performing multiple rounds of “calculating the correlation coefficient of the correlation coefficient” on the 39 chemical elements gradually reduces the systematic errors of large-area, multi-sample sampling, and the “noise” interference during the test. Doing so ensures that the elements with a positive correlation converge. In contrast, those with a negative correlation are separated. This effectively separates “communities” based on “closeness” and “sparseness”.

#### Fast-unfolding algorithm

The Fast-Unfolding algorithm is a community detection algorithm commonly used by Gephi. Additionally, it provides a method to visualize complex networks communities. Based on the community detection algorithm of hierarchical aggregation of modularity optimization^31^, we can divide social networks into multiple small communities. In this method of maximizing the modularity, the relationships between communities are restricted by each other. The modularity function is defined in the following Eq. (3).

3 $$Q = \frac{1}{2m}\sum\limits_{i,j} {\left[ {A_{i,j} - \frac{k_{i}k_{j}}{{2m}}} \right]} \begin{array}{*{20}c} {} \\ \end{array} \delta \left( {c_{i},c_{j}} \right)\begin{array}{*{20}c} , \\ \end{array} \begin{array}{*{20}c} {} \\ \end{array} \delta \left( {c_{i},c_{j}} \right) = \left\{ {_{{0\begin{array}{*{20}c} {} & {} \\ \end{array} else}}^{{1\begin{array}{*{20}c} {\begin{array}{*{20}c} {} & {} \\ \end{array} when\begin{array}{*{20}c} {} \\ \end{array} c_{i} = c_{j}} \\ \end{array} }} } \right.$$

Here, *A* denotes the adjacency matrix and *A*_*i,j*_ is the weight of the edge between the nodes *i* and *j*. *k*_*i*_ and *k*_*j*_ denote the sum of the weights of all the edges connected to node *i* and *j,* respectively. *m* is the sum of the weights of all the edges, and *c*_*i*_ is the community of node *i*. *δ (c*_*i*_*,c*_*j*_*)* indicates whether *c*_*i*_ and *c*_*j*_ are the judgment functions of the same community or not.

#### Information entropy of the topological correlation structure

Topological correlation structure features are divided into two levels: global and local features, which signify the two different network scales. On the global level, the degree of correlation between elements is mainly explained by the clustering of elements^32^. On the other hand, at the local level, the role of the elements in the whole system is primarily defined using the connectivity and neighborhood integrity of the element nodes. The meanings of the representatives at the two levels are detailed in Table 1.

**Table 1:** Elemental topology information, hierarchical metrics, and their meanings.

Degree	Aspects	Descriptive index	Meaning of network characteristics
Whole	Clustering	The smallest path	Average of the shortest path required from a node to all other nodes
Local	Connectivity	Degree	The total number of nodes directly connected to a node
	Neighborhood integrity	Clustering coefficient	The degree of direct neighborhood structural integrity of a node

We quantify the level of order or disorder in the state of the topological correlation structure of complex networks by clustering information entropy (CIE), connectivity information entropy (AIE), and neighborhood integrity information entropy (NIE). Entropy was originally a physical quantity used in thermodynamics to express the degree of disorder in the molecular state. In 1948, Claude Elwood Shannon developed the concept of “information entropy” to describe the quantification of information uncertainty or the value of describing things^33,34^, as shown in Eq. (4):

4 $$H(p) = - \sum\limits_{i = 1}^{n} {p_{i}\log (p_{i})}$$

Here, *p*_*i*_ is the probability of the occurrence of the *i*th random event, and $\sum\nolimits_{i = 1}^{n} {p_{i} = 1}$.

1. CIE : This index directly depicts the closeness or aggregation of each node with all other nodes in a global sense. The smaller the CIE of the node *i* is, the stronger its clustering ability becomes in the entire network compared with other nodes. The CIE of a node *i* is formulized in Eq. (5) as follows:
  5 $$H_{l_{i}} = - \frac{l_{i}}{{\sum\nolimits_{i = 1}^{n} {l_{i}} }}\log \left(\frac{l_{i}}{{\sum\nolimits_{i = 1}^{n} {l_{i}} }}\right)$$
  In the above Equation*, l* means the shortest path length; $\overline{l}$ is the average of the shortest path between all pairs of nodes in the network (available through the Gephi software).
2. AIE : The degree value of the node, *k*_*i*_, is the number of direct links that the node *i* forms with the other nodes in the network. This index quantifies the degree of connectivity of the nodes in the network. It reflects the closeness of the connection between the node and the surrounding nodes, which shows the importance of the node to a certain extent^35^. The AIE of node *i* is calculated, as shown in Eq. (6).
  6 $$H_{k_{i}} = - \frac{k_{i}}{{\sum\nolimits_{i = 1}^{n} {k_{i}} }}\log \left( {\frac{k_{i}}{{\sum\nolimits_{i = 1}^{n} {k_{i}} }}} \right)$$
3. NIE : The clustering coefficient, *c* ($\stackrel{-}{c}$ means the average clustering coefficient), refers to the interconnection between the neighbor nodes of a node. This is numerically equal to the ratio of the number of edges existing between nodes directly connected to a node (called neighbors of a node) to the maximum number of edges. *c* can vary between 0 and 1. The *c* value of 1 (0) means all nodes directly connected to a node are also connected to each other (not connected to each other). The node of the clustering coefficient is obtained by the following Eq. (7):
  7 $$c_{i} = \frac{2e_{i}}{{k_{i}(k_{i} - 1)}}$$

In the above Equation, *e*_*i*_ represents the actual number of edges between neighbor nodes of node *i* and *k*_*i*_ is the degree value of the node *i*. The NIE of the node *i* is defined as per the following Eq. (8):

8 $$H_{c_{i}} = - \frac{c_{i}}{{\sum\nolimits_{i = 1}^{n} {c_{i}} }}\log \left(\frac{c_{i}}{{\sum\nolimits_{i = 1}^{n} {c_{i}} }}\right)$$

#### Specific steps to create complex networks

##### Step 1

Getting the data : The source of the original data was the stream sediment dispersion flow data, including 43,584 sampling points of 39 geochemical elements. We eliminated some problematic sampling points through data preprocessing, and 40,029 valid sampling points were retained for further analysis. The total valid data is 1,561,131. Only a part of the data has been shown in Supplementary Appendix Table I.

##### Step 2

Calculating the correlation coefficient: We use SPSS software to calculate the Person correlation coefficient. We obtain the correlation coefficient matrix [39 × 39 matrix (Supplementary Appendix X_0_, Table II)] between 39 elements. To overcome random interference in large-scale data, the “correlation coefficient of the correlation coefficient” was calculated in our second iteration, and we got *X*_1_ (Supplementary Appendix Table III), *X*_2_ (Supplementary Appendix Table IV). In this paper, the *X*_2_ matrix already meets the requirements of our third step work.

##### Step 3

Visual representation of complex networks: We use the Fast unfolding algorithm in Gephi software to visualize the *X*_2_ matrix. To better display the “principal structure” of the 39 geochemical elements, we filter out the unimportant information from the weight matrix (*X*_2_) by eliminating all values below *k* to achieve “coarse-training” and obtain a 0-n matrix *X*_2_* = (*x*_ij_*).

##### Step 4

Carry out analysis of complex networks feature. They mainly include average shortest path, degree value, clustering coefficient, CIE, AIE, and NIE.

## Results and discussion

### Establishment of the topological correlation structure of geochemical complex networks

Each chemical element is denoted as a node and the element relationship as an edge. The 39 element correlation coefficient matrix is defined as an undirected, weighted, symmetric 39 × 39 adjacency matrix*.* The Fast-Unfolding community detection algorithm is used to build the initial complex networks topology of geochemical elements in the Nanling area in the Gephi software.

**Figure 2:**
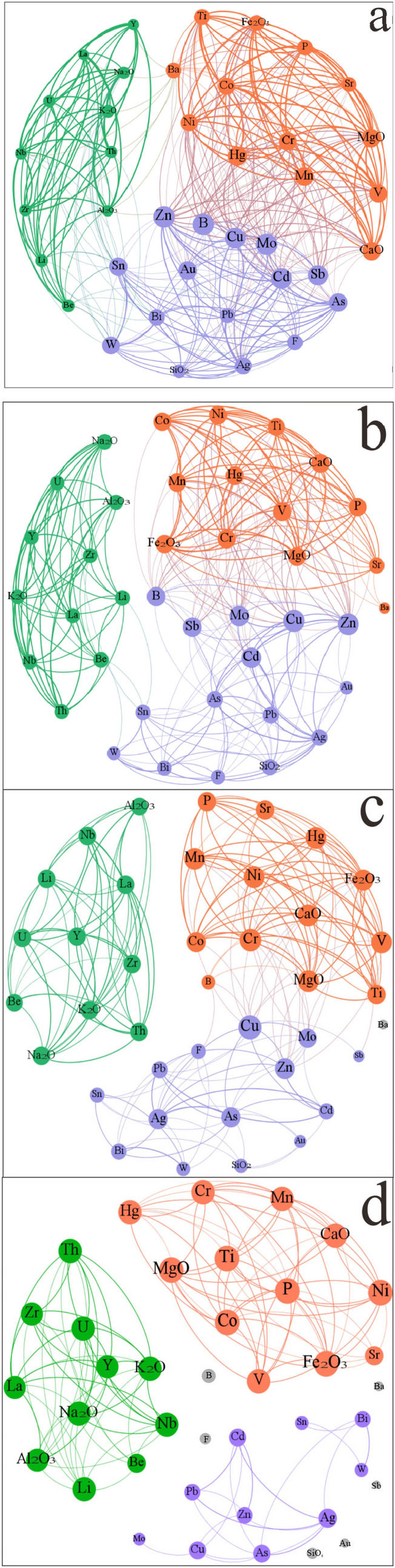
Evolution of the topological correlation structures in complex networks (control parameter, *k* = 0, 0.3, 0.5, and 0.7 from (**a**–**d**)). This figure was created with Gephi 0.9.2 (https://gephi.org).

From the topological correlation structure diagram of Fig. 2a, we deduce the following:

1. Each line indicates a positive correlation between the connected elements, and the thickness of the lines indicates the strength of positive correlation.
2. The size of each element node shows the number of edges connected to it (generally referred to as degrees).
3. There are more or less connections between any two elements. A few elements occupy the center of the topological correlation structure, while some are at the edge of the structure. Several elements act as bridges between the elements present in the middle and the elements at the edges, which present a particular community feature. The three communities are represented in the colours green (Na_2_O, K_2_O, Be, Y, Nb, U, La, Zr, Th, Al_2_O_3_, and Li), purple (Cu, Ag, Zn, Sn, Pb, As, Bi, B, W, Au, Mo, F, Cd, Sb, and SiO_2_), and orange (Fe_2_O_3_, Co, Ni, CaO, Mn, Hg, Ti, P, Sr, MgO, V, Cr, and Ba). The above elements are sorted by the topological space distance (the relationship between points and points on the network scale) from small to a large value.
4. This topological classification is consistent with the traditional elemental geochemical classification proposed by Goldschmidt. The three communities are respectively the “lithophile group” (i.e., green of Fig. 2), “chalcophile group” (i.e., purple of Fig. 2), and “siderophile group” (i.e., orange of Fig. 2). In the three communities, the green elements community was enriched mainly in acidic rocks, the purple elements community higher in hydrothermal geofluids, and the orange elements community enriched in basic rocks.
5. There are, however, some differences between them the traditional and topological classification. For example, in the conventional geochemical classification of elements, Ti, Sr, MgO, V, Mn, Cr, Ba, CaO, B, W, and SiO_2_ are lithophiles, Mo, F, Au, and Sn are siderophiles, while in the complex networks diagram, Ti, Sr, MgO, V, Mn, Cr, Ba, and CaO are siderophiles, and B, W, SiO_2_, Mo, F, Au, and Sn are chalcophiles. We explore the reasons for the discrepancy and come up with two possible answers. Firstly, the sample is derived from the stream sediments, which is quite different from the original rock. Secondly, some elements are known to exhibit duality. A transitional element can be in a symbiotic combination relationship; for instance, Mo displays the dual nature of a siderophile and chalcophile. Likewise, Mn shows the duality of belonging to both lithophile and chalcophile groups.

The classification of elements proposed by Goldschmidt is based on geochemical theory and is characterized by a strong universality. On the other hand, this work in this paper is a topological classification of actual geological bodies, which is based on the internal relationships of the geochemical element content. This classification has certain stability and is generally consistent with the Goldschmidt classification. At the same time, it also changes with the change of the research area and research object. It highlights the features arising from regionality and flexibility, which may have significance to guide prospecting. Besides, in comparison with Goldschmidt classification, the topological structure of complex networks in this paper has a smaller level of component units nested within the same type and provides a more refined internal structure. Therefore, in contrast to the Goldschmidt classification, the complex networks topological correlation structure of the geochemical field in the Nanling area may have more practical significance.

In order to demonstrate the dynamic evolution process of the topological correlation structure of complex networks, we propose a non-negative control variable *k* (element correlation coefficient). We filter out the unimportant information from the weight matrix (*X*_*2*_) by eliminating all values below *k* to achieve “coarse-graining” and obtain a 0-n matrix *X*_2_^*^ = *(x*_ij_^*^), as shown in Eq. (9):

9 $$X_{2}^{*} = \left\{ {\begin{array}{*{20}l} {x_{ij} \quad {\text{when}}\;x_{ij} > k} \hfill \\ {0\quad {\text{else}}} \hfill \\ \end{array} } \right.$$

“Coarse-graining” belongs to the idea of renormalization groups, which aims to explore the dynamic behavior of the complex system at a critical phase transition. After the “coarse-graining” of the complex networks topological correlation structure of the elements, the structure undergoes two critical phase transitions, which further resolves the community phenomena. When the control parameter (*k*) reaches 0.44, the entire element system evolves into two “communities”; when the control parameter (*k*) reaches 0.63, the system forms three stable “communities”. These “communities” are also nested inside a smaller community. Take Fig. 2d, for example: as the control parameter (*k*) becomes larger, the whole complex network structure presents smaller communities, such as W-Sn-Bi (Fig. 2d). The control parameters from a to d in Fig. 2 are 0, 0.3, 0.5, and 0.7, respectively.

### Self-organized criticality of the topological correlation structure of chemical elements

With the dynamic evolution of the complex networks’ topological correlation structure (a–d in Fig. 2), Table 2 presents trends observed in the network eigenvalues and information entropy of the topological correlation structure of geochemical elements in the Nanling area. The three sets of information entropy show an increasing trend of entropy, which reveals that the system evolves towards a dynamic equilibrium in a topological sense.

**Table 2:** Comparison of the dynamical state of topological correlation structures in complex networks in terms of entropy.

	Nodes	Edges	$\overline{c}$	$\overline{l}$	CIE	AIE	NIE
Figure 2a	39	365	0.8120	1.5990	0.1353	0.1345	0.1352
Figure 2b	39	265	0.8460	2.3600	0.1348	0.1336	0.1351
Figure 2c	38	192	0.8430	1.7830	0.1364	0.1356	0.1373
Figure 2d	33	131	0.8880	1.2950	0.1506	0.1489	0.1554

From a global perspective, the complex networks structure chart of the geochemical field in the Nanling area has a higher clustering coefficient (0.8120–0.8880) and smaller shortest path (1.2950–2.3600), which shows the small-world characteristics and reveals the self-organized criticality. From a local perspective (taking Fig. 2b as an example), there are subtle differences in the network eigenvalues and information entropy of the topological correlation structures of the geochemical elements (Table 3). Among them, the average clustering coefficient of the “lithophile group” is the highest (0.9488), while the average shortest path length of the “chalcophile group” is the lowest (1.9807). AIE, NIE, and CIE have small differences within the range of 0.05 in the three “communities”. All three indicators obey the trend of increasing entropy.

**Table 3:** Network eigenvalues and standardized information entropy of the topological correlation structures of chemical elements in the Nanling area.

Nodes	Degree	AIE	*c*	NIE	_*l*_	CIE
Ag	20	0.1422	0.5033	0.0949	1.5263	0.1310
As	22	0.1523	0.7899	0.1328	1.5000	0.1293
Au	22	0.1523	0.8009	0.1341	1.4737	0.1277
B	26	0.1714	0.7415	0.1268	1.3421	0.1192
Ba	18	0.1317	0.7582	0.1289	1.5263	0.1310
Be	14	0.1094	0.7415	0.1268	1.3421	0.1192
Bi	19	0.1370	0.7802	0.1316	1.8684	0.1516
Cd	25	0.1667	0.7567	0.1287	1.3947	0.1226
Co	20	0.1422	0.8529	0.1404	1.5526	0.1326
Cr	21	0.1473	0.5584	0.1027	1.4211	0.1243
Cu	25	0.1667	0.7802	0.1316	1.8684	0.1516
F	18	0.1317	0.8103	0.1353	1.5000	0.1293
Hg	23	0.1572	0.5762	0.1051	1.4474	0.1260
La	11	0.0912	0.6491	0.1149	1.5000	0.1293
Li	14	0.1094	0.8009	0.1341	1.4737	0.1277
Mn	22	0.1523	0.9412	0.1507	1.6316	0.1375
Mo	26	0.1714	0.7415	0.1268	1.3421	0.1192
Nb	13	0.1035	0.7567	0.1287	1.3947	0.1226
Ni	20	0.1422	0.8897	0.1448	1.6053	0.1359
P	18	0.1317	0.8316	0.1379	1.5263	0.1310
Pb	17	0.1263	0.8590	0.1411	1.7105	0.1423
Sb	24	0.1620	0.9412	0.1507	1.6316	0.1375
Sn	22	0.1523	0.8442	0.1394	1.5263	0.1310
Sr	17	0.1263	0.9273	0.1491	2.0263	0.1606
Th	13	0.1035	0.8939	0.1453	1.7368	0.1439
Ti	18	0.1317	0.8590	0.1411	1.7105	0.1423
U	13	0.1035	0.8762	0.1432	1.5526	0.1326
V	21	0.1473	0.9000	0.1460	1.5789	0.1343
W	21	0.1473	0.8590	0.1411	1.9474	0.1562
Y	12	0.0974	0.8590	0.1411	1.7105	0.1423
Zn	26	0.1714	0.8762	0.1432	1.5526	0.1326
Zr	13	0.1035	0.9000	0.1460	1.5789	0.1343
Al_2_O_3_	12	0.0974	0.8022	0.1343	1.6579	0.1391
CaO	22	0.1523	0.9412	0.1507	1.6316	0.1375
Fe_2_O_3_	18	0.1317	0.8398	0.1388	1.4737	0.1277
K_2_O	14	0.1094	0.8590	0.1411	1.9474	0.1562
MgO	22	0.1523	0.7879	0.1326	1.8684	0.1516
Na_2_O	13	0.1035	0.8485	0.1399	1.6316	0.1375
SiO_2_	15	0.1152	0.9429	0.1509	1.6579	0.1391

### Topological classification of geochemical elements and prospecting prediction

In this paper, the geochemical elements in the Nanling area are classified into three categories based on the complex networks’ topology. The three classifications are “lithophile group” (Na_2_O, K_2_O, Be, Y, Nb, U, La, Zr, Th, Al_2_O_3_, and Li), “chalcophile group” (Cu, Ag, Zn, Sn, Pb, As, Bi, B, W, Au, Mo, F, Cd, Sb, and SiO_2_) and “siderophile group” (Fe_2_O_3_, Co, Ni, CaO, Mn, Hg, Ti, P, Sr, MgO, V, Cr, and Ba), respectively. The topological classification of geochemical elements is a preliminary attempt at the practical application of complexity science in geoscience to enhance the exploration value.

Among the chalcophile elements, the group Cu–Pb–Zn–Ag exhibits a strong topological correlation structure whose enrichment is related to the mineralization of medium-acid magmatic rocks in the medium-to-low temperature environment. The topological correlation structure of W-Sn-Bi indicates that the ore-forming process is dependent on the environment. These three hydrothermal mineralization elements are related to the acidic rock mass^36^. They are abundant in the Nanling area, and the distribution direction of these elements is related to that of the Yanshan early granite. They are basically in the same extension direction of the Nanling geotectonic belt. The combination of Cu, Pb, Zn, Ag, Mo, etc., is generally associated with medium-temperature and medium–high temperature hydrothermal mineralization, which is a sign for searching hydrothermal bismuth metal mineralization. The paragenesis of Ti, Mn, and V generally signals the presence of sedimentary deposits. The elements of Cu, Mo, Au, Pb, Zn, Ag, W, etc., together with Pb, Zn, Ag, Ti, and W, are the indicators for the exploration of porphyry copper-molybdenum deposits. W, Sn, Mo, F, and Nb are often associated with alkali granites containing Na–Ta and Sn–W mineralization, sometimes with Zr, P, Ti, and Zn, which is an indication of searching for Sn, W and Nb–Ta deposits.

Among the siderophile elements, the elements of Fe, Co, and Ni are compatible. Their oxides and sulfides have low free energy, which is beneficial for enrichment under high pressure. During the historical multi-stage tectonic or magmatic activity in the Nanling area, the Yanshanian tectonic magmatism dominated and led to massive metal accumulation and mineralization^37^. Mg, Fe, Ni, Cr, Co, and B are symbiotic, closely related to ultramafic rocks, and are an important indicator for exploring iron and copper-nickel ore.

Among the lithophile elements, the properties of the elements of Be, Sr, and Ba such as an oxidation state of  (+2), quick reaction with oxygen and water vapor in the air to form oxides and carbonates on the surface, and strong alkaline water-soluble oxides, are identical to alkaline earth metals. The elements of Zr, Nb, U, La, Y, and K are incompatible, so they often appear together. The affinity of U, Y, K, Zr, Na, Nb, and La is strong, indicating high-potassium alkaline rock and K-feldspar granite with radioactive elements and local mineralization characteristics.

The Nanling area is an important non-ferrous polymetallic metallogenic area in China, and its metallogenic background and mineral prediction are the key issues that invite great attention from the geological community. In this paper, we reveal the topological structure of the regional geochemical field through the application of complex networks theory. In the future, it would be possible to make more accurate scientific judgments on the complex geological characteristics of mineralization in Nanling area of China by combining the tectonic theory of mantle plume or by using the comparison method of Th/U, Nb/Ta, Zr/Hf, and other elements. The results of the topological correlation structures in complex networks are valuable for regional prospecting prediction. This study can be further extended to process other scientifically relevant geochemical data.

## Conclusions

Based on the complex networks’ topology analysis of 39 chemical elements in the Nanling area, we have drawn the following conclusions:

1. The average clustering coefficient of the topological correlation structures for the geochemical elements in the Nanling area is between 0.8120 and 0.8880, and the average shortest path length is between 1.2950 and 2.3600. High clustering coefficients and small shortest paths indicate small-world characteristics and reveal the self-organized criticality of the geochemical field in the Nanling area.
2. Any two elements in the topological correlation structures of the geochemical elements are related to each other, but the clustering coefficient is quite different, showing certain community characteristics. On changing the control coefficient (*k)* of “coarse-graining”, the topological correlation structures display two critical phases. When the coarsening parameter (*k*) reaches 0.44, the system evolves into two parts; and when the coarsening parameter (*k*) reaches 0.63, the system forms three stable “communities”.
3. The three elemental “communities” in the sense of topology are respectively (sorted by the topological space distance from small to large): (1) Na_2_O, K_2_O, Be, Y, Nb, U, La, Zr, Th, Al_2_O_3,_ and Li; (2) Fe_2_O_3_, Co, Ni, CaO, Mn, Hg, Ti, P, Sr, MgO, V, Cr, and Ba; (3) Cu, Ag, Zn, Sn, Pb, As, Bi, B, W, Au, Mo, F, Cd, Sb, and SiO_2_, which is consistent with the geochemical classification of traditional elements proposed by Goldschmidt (“lithophile group,” “siderophile group” and “chalcophile group”). In the three communities, the green elements community was enriched mainly in acidic rocks, the purple elements community higher in hydrothermal geofluids, and the orange elements community enriched in basic rocks. As the control parameter (*k*) becomes larger, the whole complex networks structure will present smaller communities. By contrast with the traditional Goldschmidt classification, complex networks topology classification may be a more practical reference.
4. The complex networks method provides a new perspective for studying the features of chemical elements and the constraints of chemical processes. Since the results obtained from this method are consistent with the ones from traditional geochemical theory, it paves the way for the future development of the mechanism of geochemical evolution. Additionally, the complex networks theory can be applied to the study of the element migration and enrichment in time and space and the prediction of mineralization in the geological body.

## Perspectives for future works

In this paper, we take the geochemical elements in the Nanling area as the research object and demonstrate the use of the complex networks theory, a sparsely utilized method in geology. Here, we construct the complex network topology correlation structures diagram of 39 major geochemical elements. This work is based on the topological classification of actual geological bodies. It is established based on the intrinsic relationship of the content of geochemical elements. It has a certain degree of stability and is generally consistent with the Goldschmidt classification. The complex network topology structure in this paper has a certain level of the hierarchy, that is, smaller levels of constituent units are nested within the same type, which has a more refined internal structure. In the future, we introduce and apply network analysis to topics in mineralogy and petrology-fields that are especially amenable to this approach as they consider systems of numerous mineral species that coexist in myriad combinations in varied deposits. The network analysis can help to reveal the regularity and simplicity of mineralogy and petrology.

## Supplementary information

Supplementary Information.

## References

[1] Duan ZX (2019). Research on consistency control of individual behavior in complex network. Comput. Technol. Dev..

[2] Watts DJ, Strogatz SH (1998). Collective dynamics of “small-world” network. Nature.

[3] Barabasi AL, Albert R (1999). Emergence of scaling in random networks. Science.

[4] Wu XW, Qu YG, Li L (2014). River network modeling and analysis based on complex network theory. J. Hehai Univ. (Nat. Sci.).

[5] Fang K, Sivakumar B, Woldemeskel FM (2016). Complex networks, community structure, and catchment classification in a large-scale river basin. J. Hydrol..

[6] Montoya JM, Solé RV (2002). Small world patterns in food webs. J. Theor. Biol..

[7] Guo JX, Jiang YB, Xiang YB, He FJ (2017). Status analysis and countermeasures of logistics network in Ningxia based on complex network theory. Sci. Technol. Vis..

[8] Deng JL, Chen GR (2011). Research on logistics modeling method based on complex network theories. Appl. Mech. Mater..

[9] Liu HF, Qian ZY, Xu JT (2018). Research on financial index tracking optimization from the perspective of complex network. Oncol. Econ. Probl..

[10] Gao CX, Su B, Sun M, Zhang XL, Zhang ZG (2018). Interprovincial transfer of embodied primary energy in China: A complex network approach. Appl. Energy.

[11] Albert R, Barabási AL (2002). Statistical mechanics of complex networks. Rev. Mod. Phys..

[12] Che H, Gu JF (2004). Scale-free networks and their significance for systems science. Syst. Eng..

[13] Dorogovtsev SN, Mendes JFF (2010). Evolution of networks. Adv. Phys..

[14] Newman MEJ (2003). The structure and function of complex networks. SIAM Rev..

[15] Zhang BT, Wu JQ, Ling HF, Chen PR (2012). Indosinian emplacement of the Huashan-Guposhan granite batholiths in western Nanling range: Evidence from cooling-crystallization and radiogenic heat calculation of granite melt. Acta Geogr. Sin..

[16] Mao JW, Cheng YB, Chen MH, Pirajno F (2013). Major types and time-space distribution of Mesozoic ore deposits in South China and their geodynamic settings. Miner. Depos..

[17] Guo NX, Lü XQ, Zhao Z, Chen ZY (2014). Petrological and mineralogical characteristics of two types of metallogenic granitoid formed during the Mesozoic period, Nanling region. Acta Geol. Sin..

[18] Sun L, Xiao KY, Xing SW, Ding JH (2016). Characteristics and mineral potential of Nanling W-Sn-REE metallogentic belt. Acta Geol. Sin..

[19] Han K (2018). Analysis of mineral resources exploration and exploitation and utilization in Nanling district. Miner. Resour..

[20] Zhang R (2015). Constraints of in situ zircon and cassiterite U-Pb, molybdenite Re-Os and muscovite ^40^Ar-^39^Ar ages on multiple generations of granitic magmatism and related W-Sn mineralization in the Wangxianling area, Nanling Range, South China. Ore Geol. Rev..

[21] Yu CW (2011). The characteristic target-pattern regional ore zonality of the Nanling region, China (I). Geosci. Front..

[22] Yu CW (2011). The characteristic target-pattern regional ore zonality of the Nanling region, China (II). Geosci. Front..

[23] Yu CW (2011). The characteristic target-pattern regional ore zonality of the Nanling region, China (III). Geosci. Front..

[24] Yu CW (2002). Complexity of geosystem: Basic issues of geological science (I). Earth Sci..

[25] Yu CW (2003). Complexity of geosystem: Basic issues of geological science (II). Earth Sci..

[26] Wang DH, Chen YC, Wang RJ, Huang F, Wang YL (2013). Discussion on some problems related to prospecting breakthrough in Nanling region. Miner. Depos..

[27] Mao JW, Chen MH, Yuan SD, Guo CL (2011). Geological characteristics of the Qinhang (or Shihang) metallogenic belt in South China and spatial-temporal distribution regularity of mineral deposits. Acta Geol. Sin..

[28] Chen XQ, Fu JM (2012). Geochemical Atlas of Nanling Area.

[29] Chen LZ (2016). Geochemical characteristics of stream sediment survey and prospecting prediction of Shixing area, Northern Guangdong. Acta Geol. Sin..

[30] Fang JQ, Wang XF, Zheng ZG, Bi J, Di ZR (2007). New interdisciplinary science: Network science (I). Prog. Phys..

[31] Zhan WW, Xi JG, Wang ZX (2017). Hierarchical agglomerative community detection algorithm based on similarity modularity. J. Syst. Simul..

[32] Zhang H (2017). Quantitative measurements on topological structural information of road networks based on complex network analyses. Geograph. Geo-Inform. Sci..

[33] Pierce JR (1993). Looking back-Claude Elwood Shannon. IEEE Potent..

[34] Mohajeri A, Alipour M (2009). Shannon information entropy of fractional occupation probability as an electron correlation measure in atoms and molecules. Chem. Phys..

[35] Zhou T, Bai WJ, Wang BH, Liu ZJ, Yan G (2005). A brief review of complex networks. Physics.

[36] Wang DG (2014). Discussion on metallogenic specialization of the magmatic rocks and related Issues in the Nanling region. Geotect. et Metall..

[37] Pei RF, Yue XP, Yin BC, Xiong QY (1999). The explosive anomaly of ore-forming processes and super-accumulation of metals. Miner. Depos..

