# Supplementary material for: Complex network dynamics of the topological structure in a geochemical field from the Nanling area in South China
Source: Sci Rep. 2020 Nov 13;10:19826. doi: 10.1038/s41598-020-76905-6 (PMC7666206; doi:10.1038/s41598-020-76905-6)
Supplement: Supplementary file 1 — Supplementary Information. [file 41598_2020_76905_MOESM1_ESM.docx]

Complex network dynamics of the topological structure in a geochemical field from the Nanling area in South China

Nian Peng ^1^, Xiaoyan Zhu ^1^,Yongshun Liu ^1,^*, Baofeng Nie ^1^,

Ying Cui ^1^, Qianqian Geng ^1^, Chongwen Yu ^2^

^1^ College of Resource, Environment and Tourism, Capital Normal University, Beijing, 100048, China; ^2^School of Earth Sciences and Resources, China University of Geosciences (Beijing), Beijing,100083, China.

Email:

Nian Peng: 5504@cnu.edu.cn

Xiaoyan Zhu: 2398588841@qq.com

Baofeng Nie: niebaofeng@sina.com

Ying Cui: 1789214246@qq.com

Qianqian Geng: 2170902010@cnu.edu.com

Chongwen Yu: 87290809@qq.com

*Corresponding Author：Dr. Yongshun Liu, College of Resource, Environment and Tourism, Capital Normal University, 105# West Third Ring North Road, Beijing, 100048, China,. Tel: 151 1015 1299 ; E-mail: [cnu901@126.com](mailto:cnu901@126.com)

Appendices

Table Ⅰ：Source data

|  | Ag | As | Au | B | Ba | Be | Bi | Cd | Co | Cr | Cu | F | Hg | La | Li | Mn | Mo | ... |
| --- | --- | --- | --- | --- | --- | --- | --- | --- | --- | --- | --- | --- | --- | --- | --- | --- | --- | --- |
| 1 | 49.00 | 10.60 | 1.20 | 85.00 | 520.00 | 4.40 | 0.30 | 92.00 | 14.30 | 42.00 | 25.00 | 346.00 | 65.00 | 64.00 | 47.00 | 630.00 | 0.50 | ... |
| 2 | 45.00 | 15.70 | 1.70 | 41.00 | 670.00 | 3.20 | 0.60 | 148.00 | 13.20 | 32.30 | 19.00 | 456.00 | 81.00 | 102.00 | 42.00 | 545.00 | 0.60 | ... |
| 3 | 93.00 | 19.30 | 2.20 | 56.00 | 720.00 | 4.70 | 0.40 | 170.00 | 12.50 | 31.00 | 17.20 | 384.00 | 140.00 | 96.00 | 55.00 | 680.00 | 0.80 | ... |
| 4 | 60.00 | 15.80 | 1.40 | 55.00 | 450.00 | 4.00 | 0.50 | 170.00 | 16.50 | 22.00 | 15.10 | 332.00 | 45.00 | 78.00 | 54.00 | 540.00 | 0.60 | ... |
| 6 | 68.00 | 14.50 | 3.00 | 80.00 | 300.00 | 2.00 | 0.40 | 170.00 | 12.00 | 22.00 | 14.30 | 399.00 | 38.00 | 69.00 | 75.00 | 860.00 | 0.50 | ... |
| 7 | 90.00 | 14.50 | 2.80 | 91.00 | 540.00 | 3.20 | 0.50 | 150.00 | 10.00 | 18.00 | 9.90 | 399.00 | 45.00 | 56.00 | 72.00 | 680.00 | 0.70 | ... |
| 8 | 105.00 | 14.50 | 2.70 | 125.00 | 672.00 | 3.10 | 0.50 | 140.00 | 8.30 | 19.00 | 10.80 | 408.00 | 58.00 | 61.00 | 73.00 | 500.00 | 0.30 | ... |
| 9 | 80.00 | 9.00 | 0.20 | 66.00 | 440.00 | 4.00 | 0.40 | 160.00 | 6.40 | 25.50 | 4.00 | 542.00 | 50.00 | 50.00 | 76.00 | 360.00 | 0.50 | ... |
| 10 | 110.00 | 7.80 | 1.70 | 44.00 | 528.00 | 3.50 | 0.30 | 120.00 | 6.40 | 28.00 | 3.90 | 323.00 | 55.00 | 34.00 | 61.00 | 470.00 | 0.40 | ... |
| 11 | 93.00 | 6.00 | 1.30 | 56.00 | 464.00 | 2.90 | 0.30 | 280.00 | 9.00 | 35.00 | 7.70 | 371.00 | 113.00 | 44.00 | 40.00 | 900.00 | 0.50 | ... |
| 12 | 135.00 | 18.50 | 1.80 | 63.00 | 496.00 | 1.80 | 0.40 | 163.00 | 9.00 | 46.00 | 16.50 | 340.00 | 70.00 | 43.00 | 36.00 | 820.00 | 0.60 | ... |
| 13 | 49.00 | 15.50 | 1.30 | 155.00 | 450.00 | 2.00 | 0.40 | 64.00 | 5.40 | 30.00 | 13.50 | 370.00 | 40.00 | 38.50 | 19.00 | 280.00 | 0.30 | ... |
| 14 | 60.00 | 12.50 | 1.70 | 160.00 | 520.00 | 2.40 | 0.40 | 80.00 | 9.40 | 43.00 | 17.50 | 399.00 | 59.00 | 49.00 | 20.00 | 460.00 | 0.30 | ... |
| 15 | 30.00 | 10.50 | 1.10 | 53.00 | 520.00 | 2.00 | 0.30 | 82.00 | 10.00 | 55.00 | 23.00 | 342.00 | 60.00 | 38.00 | 18.00 | 420.00 | 0.60 | ... |
| 16 | 38.00 | 11.80 | 1.30 | 41.00 | 470.00 | 1.80 | 0.40 | 100.00 | 9.00 | 42.00 | 18.50 | 332.00 | 53.00 | 39.00 | 19.00 | 320.00 | 0.90 | ... |
| 17 | 72.00 | 7.20 | 22.00 | 62.00 | 530.00 | 2.40 | 0.20 | 125.00 | 10.00 | 40.00 | 20.00 | 361.00 | 68.00 | 39.50 | 29.00 | 600.00 | 0.60 | ... |
| 18 | 86.00 | 9.50 | 1.00 | 63.00 | 660.00 | 2.60 | 0.30 | 230.00 | 13.20 | 60.00 | 23.50 | 399.00 | 67.00 | 52.00 | 26.00 | 410.00 | 0.50 | ... |
| 19 | 140.00 | 3.90 | 2.00 | 69.00 | 680.00 | 3.20 | 0.20 | 490.00 | 14.00 | 77.00 | 26.50 | 333.00 | 115.00 | 53.00 | 35.00 | 540.00 | 1.30 | ... |
| 20 | 83.00 | 9.00 | 2.00 | 68.00 | 620.00 | 2.40 | 0.50 | 290.00 | 12.30 | 76.00 | 29.50 | 390.00 | 100.00 | 42.00 | 29.00 | 650.00 | 1.10 | ... |
| ... | ... | ... | ... | ... | ... | ... | ... | ... | ... | .... | ... | ... | ... | ... | ... | ... | ... | .. |

**Table Ⅱ**: Pearson correlation coefficients of the 39 elements in the Nanling area（*X_0_*）

|  | Ag | As | Au | B | Ba | Be | Bi | Cd | Co | Cr | Cu | F | Hg | La | Li | Mn | Mo | Nb | Ni |
| --- | --- | --- | --- | --- | --- | --- | --- | --- | --- | --- | --- | --- | --- | --- | --- | --- | --- | --- | --- |
| Ag | 1.0000 | 0.4195 | 0.1388 | 0.0721 | 0.0342 | 0.0601 | 0.2308 | 0.5455 | 0.0233 | 0.0073 | 0.2769 | 0.1929 | 0.0815 | -0.0028 | 0.1084 | 0.1254 | 0.2292 | -0.0055 | 0.0382 |
| As | 0.4195 | 1.0000 | 0.2291 | 0.0549 | 0.0011 | 0.0460 | 0.1760 | 0.3199 | 0.0526 | 0.0368 | 0.2107 | 0.2113 | 0.0592 | -0.0134 | 0.1082 | 0.0999 | 0.0998 | 0.0012 | 0.0576 |
| Au | 0.1388 | 0.2291 | 1.0000 | -0.0071 | 0.0239 | -0.0018 | 0.0329 | 0.0620 | 0.0345 | 0.0189 | 0.0331 | 0.0039 | 0.1467 | -0.0085 | -0.0128 | 0.0190 | 0.0394 | -0.0134 | 0.0193 |
| B | 0.0721 | 0.0549 | -0.0071 | 1.0000 | -0.0463 | 0.0543 | 0.0738 | 0.0325 | 0.0339 | 0.0810 | 0.0339 | 0.1226 | 0.0442 | -0.0724 | 0.2653 | 0.0085 | 0.0154 | -0.1071 | 0.0374 |
| Ba | 0.0342 | 0.0011 | 0.0239 | -0.0463 | 1.0000 | -0.0082 | -0.0299 | 0.0415 | 0.1416 | 0.0034 | 0.0445 | -0.0323 | 0.0018 | 0.1572 | -0.0626 | 0.0514 | 0.0906 | -0.0239 | 0.0213 |
| Be | 0.0601 | 0.0460 | -0.0018 | 0.0543 | -0.0082 | 1.0000 | 0.0650 | 0.0365 | -0.0685 | -0.0898 | 0.0146 | 0.1151 | -0.0117 | 0.0474 | 0.2155 | 0.0024 | 0.0361 | 0.1028 | -0.0431 |
| Bi | 0.2308 | 0.1760 | 0.0329 | 0.0738 | -0.0299 | 0.0650 | 1.0000 | 0.1572 | 0.0012 | -0.0330 | 0.2743 | 0.0992 | -0.0115 | 0.0073 | 0.0880 | 0.0558 | 0.3151 | 0.0436 | -0.0188 |
| Cd | 0.5455 | 0.3199 | 0.0620 | 0.0325 | 0.0415 | 0.0365 | 0.1572 | 1.0000 | 0.1117 | 0.0855 | 0.2762 | 0.1467 | 0.1808 | 0.0123 | 0.0749 | 0.1800 | 0.1848 | -0.0070 | 0.1574 |
| Co | 0.0233 | 0.0526 | 0.0345 | 0.0339 | 0.1416 | -0.0685 | 0.0012 | 0.1117 | 1.0000 | 0.6527 | 0.1559 | 0.0371 | 0.1957 | 0.0647 | -0.0158 | 0.4303 | 0.1193 | -0.0816 | 0.6867 |
| Cr | 0.0073 | 0.0368 | 0.0189 | 0.0810 | 0.0034 | -0.0898 | -0.0330 | 0.0855 | 0.6527 | 1.0000 | 0.1284 | 0.0452 | 0.2363 | -0.0615 | -0.0543 | 0.3395 | 0.1325 | -0.1763 | 0.7125 |
| Cu | 0.2769 | 0.2107 | 0.0331 | 0.0339 | 0.0445 | 0.0146 | 0.2743 | 0.2762 | 0.1559 | 0.1284 | 1.0000 | 0.0930 | 0.0650 | -0.0242 | 0.0441 | 0.1151 | 0.2154 | -0.0352 | 0.1806 |
| F | 0.1929 | 0.2113 | 0.0039 | 0.1226 | -0.0323 | 0.1151 | 0.0992 | 0.1467 | 0.0371 | 0.0452 | 0.0930 | 1.0000 | 0.0402 | 0.0007 | 0.2920 | 0.0550 | 0.0750 | 0.0430 | 0.0614 |
| Hg | 0.0815 | 0.0592 | 0.1467 | 0.0442 | 0.0018 | -0.0117 | -0.0115 | 0.1808 | 0.1957 | 0.2363 | 0.0650 | 0.0402 | 1.0000 | 0.0100 | 0.0292 | 0.1182 | 0.0602 | -0.0016 | 0.2268 |
| La | -0.0028 | -0.0134 | -0.0085 | -0.0724 | 0.1572 | 0.0474 | 0.0073 | 0.0123 | 0.0647 | -0.0615 | -0.0242 | 0.0007 | 0.0100 | 1.0000 | 0.1370 | 0.0789 | 0.0314 | 0.4606 | 0.0058 |
| Li | 0.1084 | 0.1082 | -0.0128 | 0.2653 | -0.0626 | 0.2155 | 0.0880 | 0.0749 | -0.0158 | -0.0543 | 0.0441 | 0.2920 | 0.0292 | 0.1370 | 1.0000 | 0.0642 | 0.0630 | 0.2719 | 0.0829 |
| Mn | 0.1254 | 0.0999 | 0.0190 | 0.0085 | 0.0514 | 0.0024 | 0.0558 | 0.1800 | 0.4303 | 0.3395 | 0.1151 | 0.0550 | 0.1182 | 0.0789 | 0.0642 | 1.0000 | 0.1635 | 0.0252 | 0.4896 |
| Mo | 0.2292 | 0.0998 | 0.0394 | 0.0154 | 0.0906 | 0.0361 | 0.3151 | 0.1848 | 0.1193 | 0.1325 | 0.2154 | 0.0750 | 0.0602 | 0.0314 | 0.0630 | 0.1635 | 1.0000 | 0.0540 | 0.1918 |
| Nb | -0.0055 | 0.0012 | -0.0134 | -0.1071 | -0.0239 | 0.1028 | 0.0436 | -0.0070 | -0.0816 | -0.1763 | -0.0352 | 0.0430 | -0.0016 | 0.4606 | 0.2719 | 0.0252 | 0.0540 | 1.0000 | -0.0640 |
| Ni | 0.0382 | 0.0576 | 0.0193 | 0.0374 | 0.0213 | -0.0431 | -0.0188 | 0.1574 | 0.6867 | 0.7125 | 0.1806 | 0.0614 | 0.2268 | 0.0058 | 0.0829 | 0.4896 | 0.1918 | -0.0640 | 1.0000 |
| P | 0.0468 | 0.0333 | 0.0149 | 0.0970 | 0.2161 | 0.0587 | 0.0051 | 0.1011 | 0.4069 | 0.2840 | 0.0949 | 0.0313 | 0.1396 | 0.1550 | 0.1430 | 0.2499 | 0.1219 | 0.0409 | 0.3665 |
| Pb | 0.6179 | 0.3302 | 0.1092 | 0.0136 | 0.0305 | 0.0478 | 0.2200 | 0.5375 | 0.0388 | 0.0114 | 0.2687 | 0.1335 | 0.1077 | 0.0477 | 0.0751 | 0.1698 | 0.1673 | 0.0475 | 0.0511 |
| Sb | 0.1247 | 0.1331 | 0.0213 | 0.0358 | 0.0400 | 0.0084 | 0.0310 | 0.0966 | 0.0627 | 0.0615 | 0.0662 | 0.0590 | 0.1259 | 0.0028 | 0.0248 | 0.0580 | 0.0873 | -0.0180 | 0.0643 |
| Sn | 0.1931 | 0.2991 | 0.0070 | 0.0626 | -0.0425 | 0.0756 | 0.3496 | 0.1204 | -0.0190 | -0.0398 | 0.1411 | 0.1827 | -0.0007 | 0.0191 | 0.1557 | 0.0537 | 0.0729 | 0.0920 | -0.0125 |
| Sr | 0.0010 | -0.0021 | -0.0089 | 0.0940 | 0.1483 | 0.0115 | -0.0217 | 0.0337 | 0.2014 | 0.1839 | 0.0326 | 0.0377 | 0.1011 | 0.1429 | 0.1283 | 0.1476 | 0.0243 | 0.0612 | 0.1516 |
| Th | 0.0181 | 0.0050 | -0.0002 | -0.1428 | 0.0022 | 0.0990 | 0.0757 | 0.0072 | -0.2241 | -0.3150 | -0.0414 | 0.0293 | -0.0434 | 0.5444 | 0.2576 | -0.0370 | 0.0652 | 0.6505 | -0.2000 |
| Ti | -0.0254 | 0.0026 | 0.0023 | 0.0184 | 0.0552 | -0.0692 | -0.0497 | 0.0277 | 0.5408 | 0.5378 | 0.0708 | 0.0060 | 0.1804 | 0.2075 | -0.0472 | 0.2120 | 0.0385 | 0.1222 | 0.4394 |
| U | 0.0367 | 0.0056 | -0.0118 | -0.0736 | -0.0223 | 0.1567 | 0.0857 | 0.0059 | -0.2674 | -0.3408 | -0.0392 | 0.0561 | -0.0402 | 0.4354 | 0.3565 | -0.0285 | 0.0988 | 0.6276 | -0.1944 |
| V | 0.0727 | 0.0497 | 0.0214 | 0.0760 | 0.0947 | -0.0505 | -0.0073 | 0.1159 | 0.5627 | 0.6348 | 0.1586 | 0.0513 | 0.2313 | -0.0101 | 0.0157 | 0.3378 | 0.2826 | -0.0701 | 0.6567 |
| W | 0.1944 | 0.2328 | 0.0297 | 0.0660 | -0.0166 | 0.0862 | 0.4420 | 0.1075 | -0.0137 | -0.0394 | 0.1397 | 0.1811 | 0.0047 | 0.0073 | 0.1542 | 0.0214 | 0.2255 | 0.0618 | -0.0203 |
| Y | 0.0222 | 0.0145 | -0.0114 | -0.1160 | 0.0423 | 0.0726 | 0.0284 | 0.0334 | 0.0264 | -0.0796 | -0.0033 | 0.0354 | 0.0339 | 0.5192 | 0.2445 | 0.0818 | 0.0677 | 0.5407 | 0.0426 |
| Zn | 0.3868 | 0.1947 | 0.0620 | 0.0293 | 0.0568 | 0.0269 | 0.1452 | 0.7628 | 0.1825 | 0.1503 | 0.2247 | 0.1050 | 0.2320 | 0.0550 | 0.0923 | 0.1890 | 0.1448 | 0.0395 | 0.2203 |
| Zr | -0.0413 | -0.0432 | -0.0039 | -0.1693 | 0.1169 | 0.0085 | -0.0059 | -0.0563 | -0.1185 | -0.2237 | -0.0775 | -0.0666 | -0.0709 | 0.5260 | -0.0425 | -0.0839 | -0.0481 | 0.4199 | -0.2230 |
| Al_2_O_3_ | 0.0062 | 0.0106 | -0.0042 | 0.0170 | 0.0926 | 0.1178 | 0.0473 | 0.0195 | 0.1357 | 0.0780 | 0.0243 | 0.0809 | 0.0831 | 0.4863 | 0.4442 | 0.1238 | 0.0792 | 0.4798 | 0.1729 |
| CaO | 0.0299 | 0.0426 | -0.0065 | 0.0750 | -0.1415 | -0.0126 | 0.0133 | 0.0793 | 0.1944 | 0.2584 | 0.0518 | 0.1958 | 0.1423 | -0.0865 | 0.0562 | 0.1057 | 0.0343 | -0.0535 | 0.2224 |
| Fe_2_O_3_ | 0.0985 | 0.1451 | 0.0319 | 0.0453 | 0.0647 | -0.0323 | 0.1584 | 0.1411 | 0.6690 | 0.6621 | 0.2305 | 0.1054 | 0.2300 | 0.1337 | 0.0797 | 0.3936 | 0.2210 | 0.0848 | 0.6425 |
| K_2_O | -0.0035 | -0.0267 | -0.0094 | -0.0391 | 0.1755 | 0.1643 | 0.0210 | -0.0475 | -0.3059 | -0.4796 | -0.0810 | 0.0361 | -0.1269 | 0.4028 | 0.3809 | -0.1135 | -0.0401 | 0.4199 | -0.3521 |
| MgO | 0.0755 | 0.0923 | 0.0145 | 0.0413 | 0.1400 | -0.0160 | 0.0314 | 0.1133 | 0.4471 | 0.3772 | 0.1352 | 0.1252 | 0.0789 | -0.0112 | 0.1270 | 0.2000 | 0.0633 | -0.1094 | 0.3995 |
| Na_2_O | -0.0013 | -0.0083 | -0.0122 | -0.0080 | -0.0103 | 0.0890 | 0.0161 | -0.0150 | -0.1598 | -0.1971 | -0.0347 | 0.0330 | -0.0580 | 0.1087 | 0.2311 | -0.0370 | -0.0083 | 0.2225 | -0.1344 |
| SiO_2_ | -0.0511 | -0.0571 | -0.0038 | -0.0513 | -0.0775 | -0.1264 | -0.0772 | -0.0869 | -0.2994 | -0.2365 | -0.0810 | -0.1344 | -0.1472 | -0.4148 | -0.3889 | -0.2037 | -0.1249 | -0.3790 | -0.3150 |

Connected to **Table Ⅱ**

|  | Pb | Sb | Sn | Sr | Th | Ti | U | V | W | Y | Zn | Zr | Al_2_O_3_ | CaO | Fe_2_O_3_ | K_2_O | MgO | Na_2_O | SiO_2_ |
| --- | --- | --- | --- | --- | --- | --- | --- | --- | --- | --- | --- | --- | --- | --- | --- | --- | --- | --- | --- |
| Ag | 0.6179 | 0.1247 | 0.1931 | 0.0010 | 0.0181 | -0.0254 | 0.0367 | 0.0727 | 0.1944 | 0.0222 | 0.3868 | -0.0413 | 0.0062 | 0.0299 | 0.0985 | -0.0035 | 0.0755 | -0.0013 | -0.0511 |
| As | 0.3302 | 0.1331 | 0.2991 | -0.0021 | 0.0050 | 0.0026 | 0.0056 | 0.0497 | 0.2328 | 0.0145 | 0.1947 | -0.0432 | 0.0106 | 0.0426 | 0.1451 | -0.0267 | 0.0923 | -0.0083 | -0.0571 |
| Au | 0.1092 | 0.0213 | 0.0070 | -0.0089 | -0.0002 | 0.0023 | -0.0118 | 0.0214 | 0.0297 | -0.0114 | 0.0620 | -0.0039 | -0.0042 | -0.0065 | 0.0319 | -0.0094 | 0.0145 | -0.0122 | -0.0038 |
| B | 0.0136 | 0.0358 | 0.0626 | 0.0940 | -0.1428 | 0.0184 | -0.0736 | 0.0760 | 0.0660 | -0.1160 | 0.0293 | -0.1693 | 0.0170 | 0.0750 | 0.0453 | -0.0391 | 0.0413 | -0.0080 | -0.0513 |
| Ba | 0.0305 | 0.0400 | -0.0425 | 0.1483 | 0.0022 | 0.0552 | -0.0223 | 0.0947 | -0.0166 | 0.0423 | 0.0568 | 0.1169 | 0.0926 | -0.1415 | 0.0647 | 0.1755 | 0.1400 | -0.0103 | -0.0775 |
| Be | 0.0478 | 0.0084 | 0.0756 | 0.0115 | 0.0990 | -0.0692 | 0.1567 | -0.0505 | 0.0862 | 0.0726 | 0.0269 | 0.0085 | 0.1178 | -0.0126 | -0.0323 | 0.1643 | -0.0160 | 0.0890 | -0.1264 |
| Bi | 0.2200 | 0.0310 | 0.3496 | -0.0217 | 0.0757 | -0.0497 | 0.0857 | -0.0073 | 0.4420 | 0.0284 | 0.1452 | -0.0059 | 0.0473 | 0.0133 | 0.1584 | 0.0210 | 0.0314 | 0.0161 | -0.0772 |
| Cd | 0.5375 | 0.0966 | 0.1204 | 0.0337 | 0.0072 | 0.0277 | 0.0059 | 0.1159 | 0.1075 | 0.0334 | 0.7628 | -0.0563 | 0.0195 | 0.0793 | 0.1411 | -0.0475 | 0.1133 | -0.0150 | -0.0869 |
| Co | 0.0388 | 0.0627 | -0.0190 | 0.2014 | -0.2241 | 0.5408 | -0.2674 | 0.5627 | -0.0137 | 0.0264 | 0.1825 | -0.1185 | 0.1357 | 0.1944 | 0.6690 | -0.3059 | 0.4471 | -0.1598 | -0.2994 |
| Cr | 0.0114 | 0.0615 | -0.0398 | 0.1839 | -0.3150 | 0.5378 | -0.3408 | 0.6348 | -0.0394 | -0.0796 | 0.1503 | -0.2237 | 0.0780 | 0.2584 | 0.6621 | -0.4796 | 0.3772 | -0.1971 | -0.2365 |
| Cu | 0.2687 | 0.0662 | 0.1411 | 0.0326 | -0.0414 | 0.0708 | -0.0392 | 0.1586 | 0.1397 | -0.0033 | 0.2247 | -0.0775 | 0.0243 | 0.0518 | 0.2305 | -0.0810 | 0.1352 | -0.0347 | -0.0810 |
| F | 0.1335 | 0.0590 | 0.1827 | 0.0377 | 0.0293 | 0.0060 | 0.0561 | 0.0513 | 0.1811 | 0.0354 | 0.1050 | -0.0666 | 0.0809 | 0.1958 | 0.1054 | 0.0361 | 0.1252 | 0.0330 | -0.1344 |
| Hg | 0.1077 | 0.1259 | -0.0007 | 0.1011 | -0.0434 | 0.1804 | -0.0402 | 0.2313 | 0.0047 | 0.0339 | 0.2320 | -0.0709 | 0.0831 | 0.1423 | 0.2300 | -0.1269 | 0.0789 | -0.0580 | -0.1472 |
| La | 0.0477 | 0.0028 | 0.0191 | 0.1429 | 0.5444 | 0.2075 | 0.4354 | -0.0101 | 0.0073 | 0.5192 | 0.0550 | 0.5260 | 0.4863 | -0.0865 | 0.1337 | 0.4028 | -0.0112 | 0.1087 | -0.4148 |
| Li | 0.0751 | 0.0248 | 0.1557 | 0.1283 | 0.2576 | -0.0472 | 0.3565 | 0.0157 | 0.1542 | 0.2445 | 0.0923 | -0.0425 | 0.4442 | 0.0562 | 0.0797 | 0.3809 | 0.1270 | 0.2311 | -0.3889 |
| Mn | 0.1698 | 0.0580 | 0.0537 | 0.1476 | -0.0370 | 0.2120 | -0.0285 | 0.3378 | 0.0214 | 0.0818 | 0.1890 | -0.0839 | 0.1238 | 0.1057 | 0.3936 | -0.1135 | 0.2000 | -0.0370 | -0.2037 |
| Mo | 0.1673 | 0.0873 | 0.0729 | 0.0243 | 0.0652 | 0.0385 | 0.0988 | 0.2826 | 0.2255 | 0.0677 | 0.1448 | -0.0481 | 0.0792 | 0.0343 | 0.2210 | -0.0401 | 0.0633 | -0.0083 | -0.1249 |
| Nb | 0.0475 | -0.0180 | 0.0920 | 0.0612 | 0.6505 | 0.1222 | 0.6276 | -0.0701 | 0.0618 | 0.5407 | 0.0395 | 0.4199 | 0.4798 | -0.0535 | 0.0848 | 0.4199 | -0.1094 | 0.2225 | -0.3790 |
| Ni | 0.0511 | 0.0643 | -0.0125 | 0.1516 | -0.2000 | 0.4394 | -0.1944 | 0.6567 | -0.0203 | 0.0426 | 0.2203 | -0.2230 | 0.1729 | 0.2224 | 0.6425 | -0.3521 | 0.3995 | -0.1344 | -0.3150 |
| P | 0.0471 | 0.0653 | 0.0001 | 0.2055 | -0.0116 | 0.2891 | 0.0052 | 0.3665 | 0.0208 | 0.0895 | 0.1491 | -0.0607 | 0.2466 | 0.0858 | 0.3876 | 0.0244 | 0.2691 | -0.0274 | -0.3826 |
| Pb | 1.0000 | 0.0837 | 0.1755 | 0.0197 | 0.0949 | 0.0041 | 0.0844 | 0.0432 | 0.1127 | 0.0466 | 0.5920 | 0.0121 | 0.0929 | 0.0356 | 0.1537 | 0.0414 | 0.0946 | 0.0103 | -0.1350 |
| Sb | 0.0837 | 1.0000 | 0.0423 | 0.0519 | 0.0158 | 0.0293 | -0.0113 | 0.0860 | 0.0426 | 0.0636 | 0.0863 | -0.0339 | 0.0087 | 0.0533 | 0.0837 | -0.0307 | 0.0564 | -0.0175 | -0.0362 |
| Sn | 0.1755 | 0.0423 | 1.0000 | -0.0186 | 0.0816 | -0.0560 | 0.1249 | -0.0300 | 0.1617 | 0.0850 | 0.1017 | -0.0022 | 0.0735 | 0.0159 | 0.1601 | 0.0596 | 0.0456 | 0.0397 | -0.0931 |
| Sr | 0.0197 | 0.0519 | -0.0186 | 1.0000 | 0.0349 | 0.1853 | 0.0053 | 0.1822 | -0.0135 | 0.0431 | 0.0673 | 0.0557 | 0.2014 | 0.2458 | 0.2600 | 0.0710 | 0.1787 | 0.0480 | -0.2530 |
| Th | 0.0949 | 0.0158 | 0.0816 | 0.0349 | 1.0000 | -0.1093 | 0.7503 | -0.2000 | 0.0841 | 0.5138 | 0.0347 | 0.4985 | 0.5203 | -0.0961 | -0.0582 | 0.5984 | -0.1608 | 0.2579 | -0.3944 |
| Ti | 0.0041 | 0.0293 | -0.0560 | 0.1853 | -0.1093 | 1.0000 | -0.1833 | 0.4862 | -0.0536 | 0.0627 | 0.1264 | 0.1525 | 0.2526 | 0.1359 | 0.6432 | -0.2954 | 0.2815 | -0.1522 | -0.3135 |
| U | 0.0844 | -0.0113 | 0.1249 | 0.0053 | 0.7503 | -0.1833 | 1.0000 | -0.1771 | 0.0849 | 0.4669 | 0.0175 | 0.3381 | 0.5211 | -0.0993 | -0.1107 | 0.6137 | -0.1669 | 0.2910 | -0.3880 |
| V | 0.0432 | 0.0860 | -0.0300 | 0.1822 | -0.2000 | 0.4862 | -0.1771 | 1.0000 | -0.0245 | -0.0394 | 0.1785 | -0.2470 | 0.1380 | 0.1781 | 0.6605 | -0.3656 | 0.3205 | -0.1707 | -0.2816 |
| W | 0.1127 | 0.0426 | 0.1617 | -0.0135 | 0.0841 | -0.0536 | 0.0849 | -0.0245 | 1.0000 | 0.0426 | 0.0561 | -0.0114 | 0.0431 | 0.0082 | 0.0350 | 0.0497 | 0.0164 | 0.0325 | -0.0532 |
| Y | 0.0466 | 0.0636 | 0.0850 | 0.0431 | 0.5138 | 0.0627 | 0.4669 | -0.0394 | 0.0426 | 1.0000 | 0.0676 | 0.3288 | 0.4389 | -0.0235 | 0.0861 | 0.3894 | -0.0237 | 0.1952 | -0.3728 |
| Zn | 0.5920 | 0.0863 | 0.1017 | 0.0673 | 0.0347 | 0.1264 | 0.0175 | 0.1785 | 0.0561 | 0.0676 | 1.0000 | -0.0311 | 0.1239 | 0.0846 | 0.2620 | -0.0401 | 0.1557 | -0.0082 | -0.1923 |
| Zr | 0.0121 | -0.0339 | -0.0022 | 0.0557 | 0.4985 | 0.1525 | 0.3381 | -0.2470 | -0.0114 | 0.3288 | -0.0311 | 1.0000 | 0.2741 | -0.1584 | -0.0579 | 0.3310 | -0.1288 | 0.0836 | -0.1639 |
| Al_2_O_3_ | 0.0929 | 0.0087 | 0.0735 | 0.2014 | 0.5203 | 0.2526 | 0.5211 | 0.1380 | 0.0431 | 0.4389 | 0.1239 | 0.2741 | 1.0000 | 0.0027 | 0.3917 | 0.5426 | 0.1430 | 0.1659 | -0.8125 |
| CaO | 0.0356 | 0.0533 | 0.0159 | 0.2458 | -0.0961 | 0.1359 | -0.0993 | 0.1781 | 0.0082 | -0.0235 | 0.0846 | -0.1584 | 0.0027 | 1.0000 | 0.2043 | -0.1920 | 0.2995 | -0.0139 | -0.2854 |
| Fe_2_O_3_ | 0.1537 | 0.0837 | 0.1601 | 0.2600 | -0.0582 | 0.6432 | -0.1107 | 0.6605 | 0.0350 | 0.0861 | 0.2620 | -0.0579 | 0.3917 | 0.2043 | 1.0000 | -0.2482 | 0.4071 | -0.1386 | -0.5238 |
| K_2_O | 0.0414 | -0.0307 | 0.0596 | 0.0710 | 0.5984 | -0.2954 | 0.6137 | -0.3656 | 0.0497 | 0.3894 | -0.0401 | 0.3310 | 0.5426 | -0.1920 | -0.2482 | 1.0000 | -0.0837 | 0.3169 | -0.3456 |
| MgO | 0.0946 | 0.0564 | 0.0456 | 0.1787 | -0.1608 | 0.2815 | -0.1669 | 0.3205 | 0.0164 | -0.0237 | 0.1557 | -0.1288 | 0.1430 | 0.2995 | 0.4071 | -0.0837 | 1.0000 | -0.0422 | -0.2638 |
| Na_2_O | 0.0103 | -0.0175 | 0.0397 | 0.0480 | 0.2579 | -0.1522 | 0.2910 | -0.1707 | 0.0325 | 0.1952 | -0.0082 | 0.0836 | 0.1659 | -0.0139 | -0.1386 | 0.3169 | -0.0422 | 1.0000 | -0.2325 |
| SiO_2_ | -0.4438 | -0.2137 | 0.0942 | -0.5562 | -0.0151 | 0.3504 | 0.1212 | -0.5420 | 0.3530 | 0.0372 | -0.4560 | -0.6027 | -0.5756 | -0.3316 | -0.6199 | 0.4950 | -0.5081 | 0.3434 | 1.0000 |

**Table Ⅲ**: Pearson correlation coefficients of 39 elements in the Nanling area（*X*_1_）

|  | Ag | As | Au | B | Ba | Be | Bi | Cd | Co | Cr | Cu | F | Hg | La | Li | Mn | Mo | Nb | Ni |
| --- | --- | --- | --- | --- | --- | --- | --- | --- | --- | --- | --- | --- | --- | --- | --- | --- | --- | --- | --- |
| Ag | 1.0000 | 0.7071 | 0.2146 | 0.0737 | -0.1103 | -0.0010 | 0.4259 | 0.8121 | -0.0874 | -0.0576 | 0.5106 | 0.2911 | 0.0748 | -0.2900 | -0.0308 | 0.0702 | 0.3322 | -0.2413 | -0.0466 |
| As | 0.7071 | 1.0000 | 0.3444 | 0.0870 | -0.1688 | -0.0182 | 0.3800 | 0.5530 | -0.0360 | -0.0034 | 0.4215 | 0.3494 | 0.0483 | -0.3131 | -0.0163 | 0.0540 | 0.1794 | -0.2521 | -0.0096 |
| Au | 0.2146 | 0.3444 | 1.0000 | -0.0496 | -0.0393 | -0.0980 | 0.0097 | 0.1194 | -0.0284 | -0.0078 | 0.0469 | -0.0583 | 0.1860 | -0.1835 | -0.1805 | -0.0489 | -0.0043 | -0.1778 | -0.0359 |
| B | 0.0737 | 0.0870 | -0.0496 | 1.0000 | -0.1773 | 0.0360 | 0.0735 | 0.0335 | 0.0979 | 0.1734 | 0.0773 | 0.2426 | 0.0815 | -0.3662 | 0.2358 | 0.0248 | -0.0073 | -0.3621 | 0.1228 |
| Ba | -0.1103 | -0.1688 | -0.0393 | -0.1773 | 1.0000 | -0.1121 | -0.2003 | -0.0657 | 0.1483 | 0.0270 | -0.0427 | -0.2410 | -0.0563 | 0.1926 | -0.1782 | 0.0505 | 0.0278 | -0.0415 | 0.0375 |
| Be | -0.0010 | -0.0182 | -0.0980 | 0.0360 | -0.1121 | 1.0000 | 0.0823 | -0.0831 | -0.3598 | -0.3702 | -0.1448 | 0.1689 | -0.2343 | 0.1359 | 0.4195 | -0.2491 | -0.0813 | 0.2572 | -0.3232 |
| Bi | 0.4259 | 0.3800 | 0.0097 | 0.0735 | -0.2003 | 0.0823 | 1.0000 | 0.2709 | -0.1857 | -0.1709 | 0.4347 | 0.1930 | -0.1576 | -0.1628 | 0.0671 | -0.0810 | 0.4662 | -0.0544 | -0.1686 |
| Cd | 0.8121 | 0.5530 | 0.1194 | 0.0335 | -0.0657 | -0.0831 | 0.2709 | 1.0000 | 0.1158 | 0.1324 | 0.5162 | 0.2052 | 0.2726 | -0.2743 | -0.0958 | 0.2359 | 0.2943 | -0.2750 | 0.1661 |
| Co | -0.0874 | -0.0360 | -0.0284 | 0.0979 | 0.1483 | -0.3598 | -0.1857 | 0.1158 | 1.0000 | 0.9505 | 0.2816 | -0.0444 | 0.4884 | -0.2122 | -0.2728 | 0.7720 | 0.2179 | -0.4212 | 0.9564 |
| Cr | -0.0576 | -0.0034 | -0.0078 | 0.1734 | 0.0270 | -0.3702 | -0.1709 | 0.1324 | 0.9505 | 1.0000 | 0.2977 | -0.0035 | 0.5342 | -0.3435 | -0.3147 | 0.7237 | 0.2409 | -0.5167 | 0.9599 |
| Cu | 0.5106 | 0.4215 | 0.0469 | 0.0773 | -0.0427 | -0.1448 | 0.4347 | 0.5162 | 0.2816 | 0.2977 | 1.0000 | 0.1453 | 0.1627 | -0.3473 | -0.1584 | 0.2664 | 0.4185 | -0.3627 | 0.3128 |
| F | 0.2911 | 0.3494 | -0.0583 | 0.2426 | -0.2410 | 0.1689 | 0.1930 | 0.2052 | -0.0444 | -0.0035 | 0.1453 | 1.0000 | -0.0155 | -0.1833 | 0.3732 | -0.0090 | 0.0666 | -0.0815 | 0.0053 |
| Hg | 0.0748 | 0.0483 | 0.1860 | 0.0815 | -0.0563 | -0.2343 | -0.1576 | 0.2726 | 0.4884 | 0.5342 | 0.1627 | -0.0155 | 1.0000 | -0.2134 | -0.1786 | 0.3713 | 0.1040 | -0.2839 | 0.5184 |
| La | -0.2900 | -0.3131 | -0.1835 | -0.3662 | 0.1926 | 0.1359 | -0.1628 | -0.2743 | -0.2122 | -0.3435 | -0.3473 | -0.1833 | -0.2134 | 1.0000 | 0.3693 | -0.1387 | -0.1591 | 0.8430 | -0.2544 |
| Li | -0.0308 | -0.0163 | -0.1805 | 0.2358 | -0.1782 | 0.4195 | 0.0671 | -0.0958 | -0.2728 | -0.3147 | -0.1584 | 0.3732 | -0.1786 | 0.3693 | 1.0000 | -0.1384 | -0.0625 | 0.5260 | -0.1934 |
| Mn | 0.0702 | 0.0540 | -0.0489 | 0.0248 | 0.0505 | -0.2491 | -0.0810 | 0.2359 | 0.7720 | 0.7237 | 0.2664 | -0.0090 | 0.3713 | -0.1387 | -0.1384 | 1.0000 | 0.2867 | -0.2727 | 0.8079 |
| Mo | 0.3322 | 0.1794 | -0.0043 | -0.0073 | 0.0278 | -0.0813 | 0.4662 | 0.2943 | 0.2179 | 0.2409 | 0.4185 | 0.0666 | 0.1040 | -0.1591 | -0.0625 | 0.2867 | 1.0000 | -0.1420 | 0.2862 |
| Nb | -0.2413 | -0.2521 | -0.1778 | -0.3621 | -0.0415 | 0.2572 | -0.0544 | -0.2750 | -0.4212 | -0.5167 | -0.3627 | -0.0815 | -0.2839 | 0.8430 | 0.5260 | -0.2727 | -0.1420 | 1.0000 | -0.4114 |
| Ni | -0.0466 | -0.0096 | -0.0359 | 0.1228 | 0.0375 | -0.3232 | -0.1686 | 0.1661 | 0.9564 | 0.9599 | 0.3128 | 0.0053 | 0.5184 | -0.2544 | -0.1934 | 0.8079 | 0.2862 | -0.4114 | 1.0000 |
| P | -0.1277 | -0.1236 | -0.0987 | 0.1188 | 0.3071 | -0.1245 | -0.1992 | 0.0426 | 0.7282 | 0.6375 | 0.1367 | -0.0540 | 0.3523 | 0.0737 | 0.0559 | 0.5842 | 0.1802 | -0.1225 | 0.7018 |
| Pb | 0.8796 | 0.5925 | 0.1645 | -0.0284 | -0.0816 | -0.0084 | 0.3740 | 0.8443 | -0.0563 | -0.0518 | 0.4801 | 0.2057 | 0.1204 | -0.1432 | -0.0083 | 0.1284 | 0.2678 | -0.1116 | -0.0198 |
| Sb | 0.1582 | 0.1773 | 0.0100 | 0.0384 | -0.0174 | -0.1086 | -0.0144 | 0.1411 | 0.0884 | 0.1170 | 0.1040 | 0.0349 | 0.1948 | -0.1884 | -0.1272 | 0.0677 | 0.0934 | -0.2098 | 0.0984 |
| Sn | 0.3489 | 0.4857 | -0.0228 | 0.0639 | -0.2374 | 0.1396 | 0.5865 | 0.1927 | -0.2322 | -0.2217 | 0.2257 | 0.3212 | -0.1678 | -0.0709 | 0.2208 | -0.1078 | 0.1040 | 0.0688 | -0.2050 |
| Sr | -0.2426 | -0.2292 | -0.1545 | 0.1059 | 0.2161 | -0.1276 | -0.2701 | -0.1173 | 0.4191 | 0.3857 | -0.0591 | -0.0523 | 0.2086 | 0.1380 | 0.0945 | 0.3136 | -0.0592 | -0.0133 | 0.3753 |
| Th | -0.1643 | -0.2023 | -0.1392 | -0.3684 | -0.0095 | 0.2956 | 0.0127 | -0.2354 | -0.5862 | -0.6769 | -0.3598 | -0.0725 | -0.3628 | 0.8342 | 0.5274 | -0.3926 | -0.1443 | 0.9348 | -0.5754 |
| Ti | -0.2060 | -0.1480 | -0.0755 | 0.0126 | 0.1128 | -0.3406 | -0.2632 | -0.0152 | 0.8717 | 0.8484 | 0.1361 | -0.1150 | 0.4468 | 0.0420 | -0.2431 | 0.6022 | 0.1029 | -0.1633 | 0.8193 |
| U | -0.1344 | -0.1785 | -0.1441 | -0.2911 | -0.0492 | 0.3616 | 0.0467 | -0.2246 | -0.6264 | -0.7053 | -0.3476 | -0.0179 | -0.3725 | 0.7634 | 0.6134 | -0.4031 | -0.1143 | 0.9131 | -0.5922 |
| V | -0.0270 | -0.0089 | -0.0237 | 0.1534 | 0.0910 | -0.3342 | -0.1492 | 0.1502 | 0.9248 | 0.9473 | 0.3148 | -0.0091 | 0.5263 | -0.2788 | -0.2433 | 0.7324 | 0.3576 | -0.4343 | 0.9511 |
| W | 0.3268 | 0.3813 | 0.0072 | 0.0819 | -0.1931 | 0.1507 | 0.7124 | 0.1431 | -0.2596 | -0.2426 | 0.2232 | 0.2953 | -0.1845 | -0.1295 | 0.1759 | -0.1747 | 0.3293 | -0.0008 | -0.2393 |
| Y | -0.2321 | -0.2494 | -0.1844 | -0.3824 | 0.0315 | 0.2023 | -0.0952 | -0.2351 | -0.3009 | -0.4136 | -0.3227 | -0.0980 | -0.2188 | 0.8656 | 0.4950 | -0.1677 | -0.1165 | 0.8935 | -0.2931 |
| Zn | 0.6668 | 0.4009 | 0.0811 | 0.0126 | -0.0207 | -0.1176 | 0.1896 | 0.9332 | 0.2655 | 0.2638 | 0.4643 | 0.1377 | 0.3761 | -0.1709 | -0.0636 | 0.3368 | 0.2660 | -0.2054 | 0.3084 |
| Zr | -0.2589 | -0.2802 | -0.1199 | -0.4532 | 0.1484 | 0.1331 | -0.1100 | -0.3074 | -0.4706 | -0.5737 | -0.4008 | -0.2424 | -0.3572 | 0.8404 | 0.1795 | -0.3988 | -0.2648 | 0.7959 | -0.5412 |
| Al_2_O_3_ | -0.2423 | -0.2412 | -0.1890 | -0.1676 | 0.1115 | 0.2080 | -0.1013 | -0.2010 | 0.0030 | -0.1066 | -0.2143 | -0.0020 | -0.0462 | 0.7992 | 0.6516 | 0.0524 | -0.0336 | 0.7737 | 0.0134 |
| CaO | -0.0013 | 0.0439 | -0.0498 | 0.2155 | -0.2385 | -0.1783 | -0.0807 | 0.1247 | 0.5227 | 0.5839 | 0.1710 | 0.2857 | 0.3808 | -0.3210 | -0.0443 | 0.3839 | 0.0840 | -0.3420 | 0.5524 |
| Fe_2_O_3_ | -0.0289 | 0.0276 | -0.0562 | 0.0781 | 0.0714 | -0.2978 | -0.0520 | 0.1454 | 0.9255 | 0.9054 | 0.3255 | 0.0251 | 0.4883 | -0.0696 | -0.1152 | 0.7406 | 0.3024 | -0.2320 | 0.9203 |
| K_2_O | -0.1495 | -0.1894 | -0.1248 | -0.2318 | 0.1171 | 0.3907 | 0.0015 | -0.2587 | -0.6849 | -0.7888 | -0.3851 | -0.0226 | -0.4511 | 0.7178 | 0.6193 | -0.4934 | -0.2324 | 0.8061 | -0.6924 |
| MgO | -0.0041 | 0.0448 | -0.0510 | 0.1464 | 0.1495 | -0.2626 | -0.1230 | 0.1464 | 0.8044 | 0.7743 | 0.2792 | 0.1201 | 0.3444 | -0.2722 | -0.0887 | 0.5768 | 0.1410 | -0.4377 | 0.7789 |
| Na_2_O | -0.1352 | -0.1588 | -0.1238 | -0.1417 | -0.0714 | 0.3109 | -0.0093 | -0.2122 | -0.5565 | -0.6009 | -0.3108 | 0.0127 | -0.3610 | 0.4183 | 0.5254 | -0.3863 | -0.1985 | 0.5799 | -0.5278 |
| SiO_2_ | -0.5397 | 0.0238 | 0.0881 | 0.0050 | -0.4398 | -0.4749 | -0.4170 | -0.4614 | -0.2790 | 0.1084 | -0.6000 | -0.1074 | -0.3038 | -0.9196 | -0.2528 | -0.5066 | -0.3820 | -0.3045 | -0.2901 |

Connected to **Table Ⅲ**

|  | P | Pb | Sb | Sn | Sr | Th | Ti | U | V | W | Y | Zn | Zr | Al_2_O_3_ | CaO | Fe_2_O_3_ | K_2_O | MgO | Na_2_O | SiO_2_ |
| --- | --- | --- | --- | --- | --- | --- | --- | --- | --- | --- | --- | --- | --- | --- | --- | --- | --- | --- | --- | --- |
| Ag | -0.1277 | 0.8796 | 0.1582 | 0.3489 | -0.2426 | -0.1643 | -0.2060 | -0.1344 | -0.0270 | 0.3268 | -0.2321 | 0.6668 | -0.2589 | -0.2423 | -0.0013 | -0.0289 | -0.1495 | -0.0041 | -0.1352 | 0.3255 |
| As | -0.1236 | 0.5925 | 0.1773 | 0.4857 | -0.2292 | -0.2023 | -0.1480 | -0.1785 | -0.0089 | 0.3813 | -0.2494 | 0.4009 | -0.2802 | -0.2412 | 0.0439 | 0.0276 | -0.1894 | 0.0448 | -0.1588 | 0.0762 |
| Au | -0.0987 | 0.1645 | 0.0100 | -0.0228 | -0.1545 | -0.1392 | -0.0755 | -0.1441 | -0.0237 | 0.0072 | -0.1844 | 0.0811 | -0.1199 | -0.1890 | -0.0498 | -0.0562 | -0.1248 | -0.0510 | -0.1238 | -0.1887 |
| B | 0.1188 | -0.0284 | 0.0384 | 0.0639 | 0.1059 | -0.3684 | 0.0126 | -0.2911 | 0.1534 | 0.0819 | -0.3824 | 0.0126 | -0.4532 | -0.1676 | 0.2155 | 0.0781 | -0.2318 | 0.1464 | -0.1417 | 0.2458 |
| Ba | 0.3071 | -0.0816 | -0.0174 | -0.2374 | 0.2161 | -0.0095 | 0.1128 | -0.0492 | 0.0910 | -0.1931 | 0.0315 | -0.0207 | 0.1484 | 0.1115 | -0.2385 | 0.0714 | 0.1171 | 0.1495 | -0.0714 | -0.5088 |
| Be | -0.1245 | -0.0084 | -0.1086 | 0.1396 | -0.1276 | 0.2956 | -0.3406 | 0.3616 | -0.3342 | 0.1507 | 0.2023 | -0.1176 | 0.1331 | 0.2080 | -0.1783 | -0.2978 | 0.3907 | -0.2626 | 0.3109 | 0.3489 |
| Bi | -0.1992 | 0.3740 | -0.0144 | 0.5865 | -0.2701 | 0.0127 | -0.2632 | 0.0467 | -0.1492 | 0.7124 | -0.0952 | 0.1896 | -0.1100 | -0.1013 | -0.0807 | -0.0520 | 0.0015 | -0.1230 | -0.0093 | 0.3280 |
| Cd | 0.0426 | 0.8443 | 0.1411 | 0.1927 | -0.1173 | -0.2354 | -0.0152 | -0.2246 | 0.1502 | 0.1431 | -0.2351 | 0.9332 | -0.3074 | -0.2010 | 0.1247 | 0.1454 | -0.2587 | 0.1464 | -0.2122 | -0.2179 |
| Co | 0.7282 | -0.0563 | 0.0884 | -0.2322 | 0.4191 | -0.5862 | 0.8717 | -0.6264 | 0.9248 | -0.2596 | -0.3009 | 0.2655 | -0.4706 | 0.0030 | 0.5227 | 0.9255 | -0.6849 | 0.8044 | -0.5565 | -0.5756 |
| Cr | 0.6375 | -0.0518 | 0.1170 | -0.2217 | 0.3857 | -0.6769 | 0.8484 | -0.7053 | 0.9473 | -0.2426 | -0.4136 | 0.2638 | -0.5737 | -0.1066 | 0.5839 | 0.9054 | -0.7888 | 0.7743 | -0.6009 | -0.5087 |
| Cu | 0.1367 | 0.4801 | 0.1040 | 0.2257 | -0.0591 | -0.3598 | 0.1361 | -0.3476 | 0.3148 | 0.2232 | -0.3227 | 0.4643 | -0.4008 | -0.2143 | 0.1710 | 0.3255 | -0.3851 | 0.2792 | -0.3108 | -0.4618 |
| F | -0.0540 | 0.2057 | 0.0349 | 0.3212 | -0.0523 | -0.0725 | -0.1150 | -0.0179 | -0.0091 | 0.2953 | -0.0980 | 0.1377 | -0.2424 | -0.0020 | 0.2857 | 0.0251 | -0.0226 | 0.1201 | 0.0127 | 0.2256 |
| Hg | 0.3523 | 0.1204 | 0.1948 | -0.1678 | 0.2086 | -0.3628 | 0.4468 | -0.3725 | 0.5263 | -0.1845 | -0.2188 | 0.3761 | -0.3572 | -0.0462 | 0.3808 | 0.4883 | -0.4511 | 0.3444 | -0.3610 | -0.3809 |
| La | 0.0737 | -0.1432 | -0.1884 | -0.0709 | 0.1380 | 0.8342 | 0.0420 | 0.7634 | -0.2788 | -0.1295 | 0.8656 | -0.1709 | 0.8404 | 0.7992 | -0.3210 | -0.0696 | 0.7178 | -0.2722 | 0.4183 | -0.7641 |
| Li | 0.0559 | -0.0083 | -0.1272 | 0.2208 | 0.0945 | 0.5274 | -0.2431 | 0.6134 | -0.2433 | 0.1759 | 0.4950 | -0.0636 | 0.1795 | 0.6516 | -0.0443 | -0.1152 | 0.6193 | -0.0887 | 0.5254 | 0.2669 |
| Mn | 0.5842 | 0.1284 | 0.0677 | -0.1078 | 0.3136 | -0.3926 | 0.6022 | -0.4031 | 0.7324 | -0.1747 | -0.1677 | 0.3368 | -0.3988 | 0.0524 | 0.3839 | 0.7406 | -0.4934 | 0.5768 | -0.3863 | -0.4891 |
| Mo | 0.1802 | 0.2678 | 0.0934 | 0.1040 | -0.0592 | -0.1443 | 0.1029 | -0.1143 | 0.3576 | 0.3293 | -0.1165 | 0.2660 | -0.2648 | -0.0336 | 0.0840 | 0.3024 | -0.2324 | 0.1410 | -0.1985 | 0.2679 |
| Nb | -0.1225 | -0.1116 | -0.2098 | 0.0688 | -0.0133 | 0.9348 | -0.1633 | 0.9131 | -0.4343 | -0.0008 | 0.8935 | -0.2054 | 0.7959 | 0.7737 | -0.3420 | -0.2320 | 0.8061 | -0.4377 | 0.5799 | 0.0149 |
| Ni | 0.7018 | -0.0198 | 0.0984 | -0.2050 | 0.3753 | -0.5754 | 0.8193 | -0.5922 | 0.9511 | -0.2393 | -0.2931 | 0.3084 | -0.5412 | 0.0134 | 0.5524 | 0.9203 | -0.6924 | 0.7789 | -0.5278 | -0.3415 |
| P | 1.0000 | -0.0767 | 0.0424 | -0.2111 | 0.4654 | -0.2400 | 0.6560 | -0.2476 | 0.7001 | -0.2195 | -0.0263 | 0.1967 | -0.2518 | 0.3108 | 0.3353 | 0.7274 | -0.2595 | 0.6190 | -0.2683 | -0.2934 |
| Pb | -0.0767 | 1.0000 | 0.1004 | 0.3095 | -0.1759 | -0.0420 | -0.1371 | -0.0328 | -0.0248 | 0.2092 | -0.1091 | 0.8128 | -0.1379 | -0.0813 | 0.0006 | 0.0365 | -0.0624 | 0.0190 | -0.0825 | -0.4438 |
| Sb | 0.0424 | 0.1004 | 1.0000 | -0.0080 | -0.0075 | -0.1777 | 0.0262 | -0.1896 | 0.1239 | -0.0104 | -0.1286 | 0.1169 | -0.2073 | -0.1546 | 0.1020 | 0.0769 | -0.1938 | 0.0768 | -0.1709 | -0.2137 |
| Sn | -0.2111 | 0.3095 | -0.0080 | 1.0000 | -0.2422 | 0.1040 | -0.2788 | 0.1543 | -0.2174 | 0.3809 | 0.0367 | 0.1187 | -0.0391 | 0.0093 | -0.0764 | -0.0722 | 0.1148 | -0.1238 | 0.0884 | 0.0942 |
| Sr | 0.4654 | -0.1759 | -0.0075 | -0.2422 | 1.0000 | -0.0877 | 0.4384 | -0.1194 | 0.3871 | -0.2665 | 0.0087 | 0.0061 | -0.0446 | 0.2941 | 0.4401 | 0.4515 | -0.0786 | 0.4092 | -0.0688 | -0.5562 |
| Th | -0.2400 | -0.0420 | -0.1777 | 0.1040 | -0.0877 | 1.0000 | -0.3741 | 0.9717 | -0.5953 | 0.0662 | 0.8623 | -0.2062 | 0.8287 | 0.7234 | -0.4258 | -0.4090 | 0.9102 | -0.5402 | 0.6430 | -0.0151 |
| Ti | 0.6560 | -0.1371 | 0.0262 | -0.2788 | 0.4384 | -0.3741 | 1.0000 | -0.4478 | 0.8306 | -0.3227 | -0.1112 | 0.1718 | -0.1632 | 0.1890 | 0.4267 | 0.8980 | -0.5436 | 0.6549 | -0.4786 | 0.3504 |
| U | -0.2476 | -0.0328 | -0.1896 | 0.1543 | -0.1194 | 0.9717 | -0.4478 | 1.0000 | -0.6083 | 0.1016 | 0.8285 | -0.2109 | 0.7393 | 0.7104 | -0.4226 | -0.4471 | 0.9259 | -0.5522 | 0.6815 | 0.1212 |
| V | 0.7001 | -0.0248 | 0.1239 | -0.2174 | 0.3871 | -0.5953 | 0.8306 | -0.6083 | 1.0000 | -0.2296 | -0.3472 | 0.2866 | -0.5557 | -0.0190 | 0.5219 | 0.9185 | -0.7146 | 0.7394 | -0.5654 | -0.5420 |
| W | -0.2195 | 0.2092 | -0.0104 | 0.3809 | -0.2665 | 0.0662 | -0.3227 | 0.1016 | -0.2296 | 1.0000 | -0.0474 | 0.0260 | -0.0736 | -0.0826 | -0.1075 | -0.1849 | 0.0851 | -0.1823 | 0.0639 | 0.3530 |
| Y | -0.0263 | -0.1091 | -0.1286 | 0.0367 | 0.0087 | 0.8623 | -0.1112 | 0.8285 | -0.3472 | -0.0474 | 1.0000 | -0.1573 | 0.7313 | 0.7726 | -0.2893 | -0.1527 | 0.7523 | -0.3277 | 0.5294 | 0.0372 |
| Zn | 0.1967 | 0.8128 | 0.1169 | 0.1187 | 0.0061 | -0.2062 | 0.1718 | -0.2109 | 0.2866 | 0.0260 | -0.1573 | 1.0000 | -0.2686 | -0.0403 | 0.1867 | 0.3235 | -0.2628 | 0.2620 | -0.2234 | -0.4560 |
| Zr | -0.2518 | -0.1379 | -0.2073 | -0.0391 | -0.0446 | 0.8287 | -0.1632 | 0.7393 | -0.5557 | -0.0736 | 0.7313 | -0.2686 | 1.0000 | 0.5512 | -0.4768 | -0.3609 | 0.7264 | -0.4986 | 0.4345 | -0.6027 |
| Al_2_O_3_ | 0.3108 | -0.0813 | -0.1546 | 0.0093 | 0.2941 | 0.7234 | 0.1890 | 0.7104 | -0.0190 | -0.0826 | 0.7726 | -0.0403 | 0.5512 | 1.0000 | -0.0744 | 0.2242 | 0.6586 | 0.0156 | 0.4212 | -0.5756 |
| CaO | 0.3353 | 0.0006 | 0.1020 | -0.0764 | 0.4401 | -0.4258 | 0.4267 | -0.4226 | 0.5219 | -0.1075 | -0.2893 | 0.1867 | -0.4768 | -0.0744 | 1.0000 | 0.5151 | -0.4868 | 0.6210 | -0.2693 | -0.3316 |
| Fe_2_O_3_ | 0.7274 | 0.0365 | 0.0769 | -0.0722 | 0.4515 | -0.4090 | 0.8980 | -0.4471 | 0.9185 | -0.1849 | -0.1527 | 0.3235 | -0.3609 | 0.2242 | 0.5151 | 1.0000 | -0.5578 | 0.7562 | -0.4745 | -0.6199 |
| K_2_O | -0.2595 | -0.0624 | -0.1938 | 0.1148 | -0.0786 | 0.9102 | -0.5436 | 0.9259 | -0.7146 | 0.0851 | 0.7523 | -0.2628 | 0.7264 | 0.6586 | -0.4868 | -0.5578 | 1.0000 | -0.5276 | 0.7119 | 0.4950 |
| MgO | 0.6190 | 0.0190 | 0.0768 | -0.1238 | 0.4092 | -0.5402 | 0.6549 | -0.5522 | 0.7394 | -0.1823 | -0.3277 | 0.2620 | -0.4986 | 0.0156 | 0.6210 | 0.7562 | -0.5276 | 1.0000 | -0.4086 | -0.5081 |
| Na_2_O | -0.2683 | -0.0825 | -0.1709 | 0.0884 | -0.0688 | 0.6430 | -0.4786 | 0.6815 | -0.5654 | 0.0639 | 0.5294 | -0.2234 | 0.4345 | 0.4212 | -0.2693 | -0.4745 | 0.7119 | -0.4086 | 1.0000 | 0.3434 |
| SiO_2_ | -0.5397 | 0.0238 | 0.0881 | 0.0050 | -0.4398 | -0.4749 | -0.4170 | -0.4614 | -0.2790 | 0.1084 | -0.6000 | -0.1074 | -0.3038 | -0.9196 | -0.2528 | -0.5066 | -0.3820 | -0.3045 | -0.2901 | 1.0000 |

**Table Ⅳ：**Pearson correlation coefficient of 39 kinds of elements in the Nanling area（）

|  | Ag | As | Au | B | Ba | Be | Bi | Cd | Co | Cr | Cu | F | Hg | La | Li | Mn | Mo | Nb | Ni |
| --- | --- | --- | --- | --- | --- | --- | --- | --- | --- | --- | --- | --- | --- | --- | --- | --- | --- | --- | --- |
| Ag | 1.0000 | 0.9444 | 0.5242 | 0.2828 | -0.4093 | -0.1184 | 0.7210 | 0.9225 | -0.0486 | 0.0188 | 0.7386 | 0.5404 | 0.1370 | -0.5891 | -0.3173 | 0.0397 | 0.5543 | -0.4686 | -0.0112 |
| As | 0.9444 | 1.0000 | 0.6053 | 0.3661 | -0.4773 | -0.1532 | 0.7201 | 0.8438 | 0.0006 | 0.0748 | 0.7381 | 0.6143 | 0.1614 | -0.6585 | -0.3448 | 0.0670 | 0.5191 | -0.5318 | 0.0360 |
| Au | 0.5242 | 0.6053 | 1.0000 | 0.1502 | -0.1787 | -0.2682 | 0.2334 | 0.4726 | 0.0524 | 0.1058 | 0.3863 | 0.0906 | 0.2653 | -0.4958 | -0.4734 | 0.0493 | 0.2121 | -0.4440 | 0.0594 |
| B | 0.2828 | 0.3661 | 0.1502 | 1.0000 | -0.3139 | -0.2991 | 0.1400 | 0.3455 | 0.4390 | 0.5041 | 0.5018 | 0.5081 | 0.4569 | -0.7348 | -0.2929 | 0.4065 | 0.3384 | -0.7027 | 0.4656 |
| Ba | -0.4093 | -0.4773 | -0.1787 | -0.3139 | 1.0000 | -0.2475 | -0.5288 | -0.2701 | 0.2497 | 0.1746 | -0.1958 | -0.6220 | 0.0840 | 0.2492 | -0.1857 | 0.1982 | -0.1036 | 0.0602 | 0.1934 |
| Be | -0.1184 | -0.1532 | -0.2682 | -0.2991 | -0.2475 | 1.0000 | 0.2260 | -0.3766 | -0.8203 | -0.8287 | -0.5756 | 0.1801 | -0.7965 | 0.5759 | 0.8126 | -0.7946 | -0.4873 | 0.7135 | -0.8083 |
| Bi | 0.7210 | 0.7201 | 0.2334 | 0.1400 | -0.5288 | 0.2260 | 1.0000 | 0.4914 | -0.3730 | -0.3151 | 0.4642 | 0.5256 | -0.2981 | -0.2881 | 0.0367 | -0.3031 | 0.4830 | -0.1123 | -0.3392 |
| Cd | 0.9225 | 0.8438 | 0.4726 | 0.3455 | -0.2701 | -0.3766 | 0.4914 | 1.0000 | 0.2750 | 0.3302 | 0.8472 | 0.4258 | 0.4507 | -0.6822 | -0.5002 | 0.3600 | 0.6420 | -0.6319 | 0.3099 |
| Co | -0.0486 | 0.0006 | 0.0524 | 0.4390 | 0.2497 | -0.8203 | -0.3730 | 0.2750 | 1.0000 | 0.9939 | 0.5466 | -0.0873 | 0.9002 | -0.5564 | -0.6684 | 0.9779 | 0.4999 | -0.7181 | 0.9971 |
| Cr | 0.0188 | 0.0748 | 0.1058 | 0.5041 | 0.1746 | -0.8287 | -0.3151 | 0.3302 | 0.9939 | 1.0000 | 0.5997 | -0.0252 | 0.9173 | -0.6360 | -0.7062 | 0.9706 | 0.5347 | -0.7797 | 0.9955 |
| Cu | 0.7386 | 0.7381 | 0.3863 | 0.5018 | -0.1958 | -0.5756 | 0.4642 | 0.8472 | 0.5466 | 0.5997 | 1.0000 | 0.3728 | 0.5937 | -0.8247 | -0.6621 | 0.5917 | 0.8183 | -0.8140 | 0.5750 |
| F | 0.5404 | 0.6143 | 0.0906 | 0.5081 | -0.6220 | 0.1801 | 0.5256 | 0.4258 | -0.0873 | -0.0252 | 0.3728 | 1.0000 | -0.0166 | -0.4006 | 0.1696 | -0.0488 | 0.2381 | -0.2531 | -0.0408 |
| Hg | 0.1370 | 0.1614 | 0.2653 | 0.4569 | 0.0840 | -0.7965 | -0.2981 | 0.4507 | 0.9002 | 0.9173 | 0.5937 | -0.0166 | 1.0000 | -0.6330 | -0.6956 | 0.8854 | 0.4879 | -0.7526 | 0.9095 |
| La | -0.5891 | -0.6585 | -0.4958 | -0.7348 | 0.2492 | 0.5759 | -0.2881 | -0.6822 | -0.5564 | -0.6360 | -0.8247 | -0.4006 | -0.6330 | 1.0000 | 0.7615 | -0.5487 | -0.6198 | 0.9690 | -0.5798 |
| Li | -0.3173 | -0.3448 | -0.4734 | -0.2929 | -0.1857 | 0.8126 | 0.0367 | -0.5002 | -0.6684 | -0.7062 | -0.6621 | 0.1696 | -0.6956 | 0.7615 | 1.0000 | -0.6360 | -0.5190 | 0.8475 | -0.6550 |
| Mn | 0.0397 | 0.0670 | 0.0493 | 0.4065 | 0.1982 | -0.7946 | -0.3031 | 0.3600 | 0.9779 | 0.9706 | 0.5917 | -0.0488 | 0.8854 | -0.5487 | -0.6360 | 1.0000 | 0.5644 | -0.6979 | 0.9837 |
| Mo | 0.5543 | 0.5191 | 0.2121 | 0.3384 | -0.1036 | -0.4873 | 0.4830 | 0.6420 | 0.4999 | 0.5347 | 0.8183 | 0.2381 | 0.4879 | -0.6198 | -0.5190 | 0.5644 | 1.0000 | -0.6191 | 0.5320 |
| Nb | -0.4686 | -0.5318 | -0.4440 | -0.7027 | 0.0602 | 0.7135 | -0.1123 | -0.6319 | -0.7181 | -0.7797 | -0.8140 | -0.2531 | -0.7526 | 0.9690 | 0.8475 | -0.6979 | -0.6191 | 1.0000 | -0.7300 |
| Ni | -0.0112 | 0.0360 | 0.0594 | 0.4656 | 0.1934 | -0.8083 | -0.3392 | 0.3099 | 0.9971 | 0.9955 | 0.5750 | -0.0408 | 0.9095 | -0.5798 | -0.6550 | 0.9837 | 0.5320 | -0.7300 | 1.0000 |
| P | -0.2737 | -0.2504 | -0.1590 | 0.3061 | 0.4158 | -0.6579 | -0.5307 | 0.0573 | 0.9271 | 0.8880 | 0.3110 | -0.2021 | 0.7801 | -0.2667 | -0.4080 | 0.9050 | 0.3450 | -0.4592 | 0.9173 |
| Pb | 0.9710 | 0.8721 | 0.4572 | 0.1477 | -0.3513 | -0.0969 | 0.6577 | 0.9285 | -0.0630 | -0.0121 | 0.6855 | 0.4579 | 0.1308 | -0.4527 | -0.2451 | 0.0430 | 0.5056 | -0.3518 | -0.0270 |
| Sb | 0.3913 | 0.4284 | 0.2953 | 0.3757 | -0.0905 | -0.4873 | 0.0646 | 0.4803 | 0.4040 | 0.4521 | 0.5242 | 0.1572 | 0.5268 | -0.6245 | -0.5706 | 0.3984 | 0.4081 | -0.6327 | 0.4174 |
| Sn | 0.5899 | 0.6378 | 0.1284 | 0.0350 | -0.5875 | 0.4419 | 0.8609 | 0.3181 | -0.5562 | -0.5104 | 0.1965 | 0.5949 | -0.4754 | -0.0558 | 0.3149 | -0.4846 | 0.1306 | 0.1373 | -0.5236 |
| Sr | -0.5243 | -0.5037 | -0.3306 | 0.1913 | 0.4459 | -0.4858 | -0.6962 | -0.2306 | 0.7285 | 0.6797 | -0.0177 | -0.2800 | 0.5734 | -0.0119 | -0.1774 | 0.6792 | 0.0076 | -0.2038 | 0.7065 |
| Th | -0.3661 | -0.4350 | -0.3765 | -0.6905 | 0.0162 | 0.7667 | -0.0068 | -0.5706 | -0.8138 | -0.8653 | -0.7861 | -0.1970 | -0.8206 | 0.9298 | 0.8478 | -0.7892 | -0.6116 | 0.9876 | -0.8234 |
| Ti | -0.2337 | -0.1908 | -0.0644 | 0.2945 | 0.3284 | -0.7749 | -0.5145 | 0.1034 | 0.9695 | 0.9441 | 0.3724 | -0.2225 | 0.8452 | -0.3539 | -0.5637 | 0.9346 | 0.3644 | -0.5442 | 0.9577 |
| U | -0.3328 | -0.3994 | -0.3684 | -0.6503 | -0.0280 | 0.8010 | 0.0368 | -0.5520 | -0.8390 | -0.8856 | -0.7715 | -0.1437 | -0.8387 | 0.9055 | 0.8717 | -0.8109 | -0.5992 | 0.9782 | -0.8448 |
| V | 0.0041 | 0.0505 | 0.0775 | 0.4789 | 0.2042 | -0.8182 | -0.3227 | 0.3197 | 0.9960 | 0.9971 | 0.5899 | -0.0440 | 0.9125 | -0.6008 | -0.6790 | 0.9777 | 0.5542 | -0.7498 | 0.9982 |
| W | 0.5767 | 0.6022 | 0.1689 | 0.0805 | -0.5417 | 0.4200 | 0.9292 | 0.2869 | -0.5518 | -0.4977 | 0.2215 | 0.5535 | -0.4770 | -0.1317 | 0.2317 | -0.4998 | 0.2756 | 0.0643 | -0.5213 |
| Y | -0.5081 | -0.5747 | -0.4728 | -0.7223 | 0.1182 | 0.6631 | -0.1772 | -0.6415 | -0.6526 | -0.7217 | -0.8120 | -0.2965 | -0.6998 | 0.9841 | 0.8282 | -0.6312 | -0.6084 | 0.9935 | -0.6662 |
| Zn | 0.7789 | 0.6882 | 0.3774 | 0.3359 | -0.1419 | -0.5153 | 0.2909 | 0.9561 | 0.4853 | 0.5211 | 0.8415 | 0.3067 | 0.6287 | -0.6417 | -0.5404 | 0.5656 | 0.6579 | -0.6509 | 0.5157 |
| Zr | -0.4539 | -0.5226 | -0.3696 | -0.7646 | 0.1539 | 0.6710 | -0.1222 | -0.6294 | -0.7538 | -0.8141 | -0.8242 | -0.3520 | -0.7836 | 0.9541 | 0.7373 | -0.7500 | -0.6619 | 0.9734 | -0.7777 |
| Al_2_O_3_ | -0.6329 | -0.6867 | -0.5806 | -0.5797 | 0.2308 | 0.4966 | -0.3592 | -0.6564 | -0.3442 | -0.4300 | -0.7361 | -0.3047 | -0.4507 | 0.9407 | 0.7966 | -0.3305 | -0.5140 | 0.8875 | -0.3582 |
| CaO | 0.0631 | 0.1343 | 0.0680 | 0.6075 | -0.0518 | -0.7088 | -0.2460 | 0.3405 | 0.8995 | 0.9217 | 0.5705 | 0.2177 | 0.8619 | -0.6553 | -0.5579 | 0.8753 | 0.4616 | -0.7503 | 0.9132 |
| Fe_2_O_3_ | -0.0696 | -0.0247 | -0.0131 | 0.3919 | 0.2272 | -0.7815 | -0.3563 | 0.2546 | 0.9875 | 0.9738 | 0.5245 | -0.0734 | 0.8793 | -0.4713 | -0.5775 | 0.9751 | 0.5091 | -0.6347 | 0.9875 |
| K_2_O | -0.3252 | -0.3894 | -0.3467 | -0.6222 | 0.0104 | 0.8188 | 0.0348 | -0.5569 | -0.8658 | -0.9104 | -0.7824 | -0.1372 | -0.8668 | 0.8847 | 0.8648 | -0.8464 | -0.6364 | 0.9595 | -0.8751 |
| MgO | 0.0120 | 0.0689 | 0.0523 | 0.5151 | 0.2018 | -0.7847 | -0.3195 | 0.3201 | 0.9812 | 0.9812 | 0.5834 | 0.0384 | 0.8790 | -0.6071 | -0.6274 | 0.9561 | 0.4987 | -0.7510 | 0.9819 |
| Na_2_O | -0.3063 | -0.3628 | -0.3430 | -0.5532 | -0.0854 | 0.8319 | 0.0555 | -0.5427 | -0.8698 | -0.9047 | -0.7653 | -0.0706 | -0.8614 | 0.8255 | 0.8785 | -0.8479 | -0.6353 | 0.9229 | -0.8720 |
| SiO_2_ | 0.6412 | 0.6742 | 0.5924 | 0.3559 | -0.2898 | -0.1594 | 0.5095 | 0.5158 | -0.1080 | -0.0195 | 0.4822 | 0.2716 | 0.0382 | -0.7024 | -0.5614 | -0.1186 | 0.2778 | -0.5839 | -0.0982 |

Connected to **Table Ⅳ**

|  | P | Pb | Sb | Sn | Sr | Th | Ti | U | V | W | Y | Zn | Zr | Al2O3 | CaO | Fe_2_O_3_ | K_2_O | MgO | Na_2_O | SiO_2_ |
| --- | --- | --- | --- | --- | --- | --- | --- | --- | --- | --- | --- | --- | --- | --- | --- | --- | --- | --- | --- | --- |
| Ag | -0.2737 | 0.9710 | 0.3913 | 0.5899 | -0.5243 | -0.3661 | -0.2337 | -0.3328 | 0.0041 | 0.5767 | -0.5081 | 0.7789 | -0.4539 | -0.6329 | 0.0631 | -0.0696 | -0.3252 | 0.0120 | -0.3063 | 0.7725 |
| As | -0.2504 | 0.8721 | 0.4284 | 0.6378 | -0.5037 | -0.4350 | -0.1908 | -0.3994 | 0.0505 | 0.6022 | -0.5747 | 0.6882 | -0.5226 | -0.6867 | 0.1343 | -0.0247 | -0.3894 | 0.0689 | -0.3628 | 0.0788 |
| Au | -0.1590 | 0.4572 | 0.2953 | 0.1284 | -0.3306 | -0.3765 | -0.0644 | -0.3684 | 0.0775 | 0.1689 | -0.4728 | 0.3774 | -0.3696 | -0.5806 | 0.0680 | -0.0131 | -0.3467 | 0.0523 | -0.3430 | -0.5905 |
| B | 0.3061 | 0.1477 | 0.3757 | 0.0350 | 0.1913 | -0.6905 | 0.2945 | -0.6503 | 0.4789 | 0.0805 | -0.7223 | 0.3359 | -0.7646 | -0.5797 | 0.6075 | 0.3919 | -0.6222 | 0.5151 | -0.5532 | 0.6129 |
| Ba | 0.4158 | -0.3513 | -0.0905 | -0.5875 | 0.4459 | 0.0162 | 0.3284 | -0.0280 | 0.2042 | -0.5417 | 0.1182 | -0.1419 | 0.1539 | 0.2308 | -0.0518 | 0.2272 | 0.0104 | 0.2018 | -0.0854 | -0.8250 |
| Be | -0.6579 | -0.0969 | -0.4873 | 0.4419 | -0.4858 | 0.7667 | -0.7749 | 0.8010 | -0.8182 | 0.4200 | 0.6631 | -0.5153 | 0.6710 | 0.4966 | -0.7088 | -0.7815 | 0.8188 | -0.7847 | 0.8319 | 0.8319 |
| Bi | -0.5307 | 0.6577 | 0.0646 | 0.8609 | -0.6962 | -0.0068 | -0.5145 | 0.0368 | -0.3227 | 0.9292 | -0.1772 | 0.2909 | -0.1222 | -0.3592 | -0.2460 | -0.3563 | 0.0348 | -0.3195 | 0.0555 | 0.7984 |
| Cd | 0.0573 | 0.9285 | 0.4803 | 0.3181 | -0.2306 | -0.5706 | 0.1034 | -0.5520 | 0.3197 | 0.2869 | -0.6415 | 0.9561 | -0.6294 | -0.6564 | 0.3405 | 0.2546 | -0.5569 | 0.3201 | -0.5427 | -0.6526 |
| Co | 0.9271 | -0.0630 | 0.4040 | -0.5562 | 0.7285 | -0.8138 | 0.9695 | -0.8390 | 0.9960 | -0.5518 | -0.6526 | 0.4853 | -0.7538 | -0.3442 | 0.8995 | 0.9875 | -0.8658 | 0.9812 | -0.8698 | -0.8433 |
| Cr | 0.8880 | -0.0121 | 0.4521 | -0.5104 | 0.6797 | -0.8653 | 0.9441 | -0.8856 | 0.9971 | -0.4977 | -0.7217 | 0.5211 | -0.8141 | -0.4300 | 0.9217 | 0.9738 | -0.9104 | 0.9812 | -0.9047 | -0.7924 |
| Cu | 0.3110 | 0.6855 | 0.5242 | 0.1965 | -0.0177 | -0.7861 | 0.3724 | -0.7715 | 0.5899 | 0.2215 | -0.8120 | 0.8415 | -0.8242 | -0.7361 | 0.5705 | 0.5245 | -0.7824 | 0.5834 | -0.7653 | -0.7238 |
| F | -0.2021 | 0.4579 | 0.1572 | 0.5949 | -0.2800 | -0.1970 | -0.2225 | -0.1437 | -0.0440 | 0.5535 | -0.2965 | 0.3067 | -0.3520 | -0.3047 | 0.2177 | -0.0734 | -0.1372 | 0.0384 | -0.0706 | 0.7239 |
| Hg | 0.7801 | 0.1308 | 0.5268 | -0.4754 | 0.5734 | -0.8206 | 0.8452 | -0.8387 | 0.9125 | -0.4770 | -0.6998 | 0.6287 | -0.7836 | -0.4507 | 0.8619 | 0.8793 | -0.8668 | 0.8790 | -0.8614 | -0.7644 |
| La | -0.2667 | -0.4527 | -0.6245 | -0.0558 | -0.0119 | 0.9298 | -0.3539 | 0.9055 | -0.6008 | -0.1317 | 0.9841 | -0.6417 | 0.9541 | 0.9407 | -0.6553 | -0.4713 | 0.8847 | -0.6071 | 0.8255 | -0.9484 |
| Li | -0.4080 | -0.2451 | -0.5706 | 0.3149 | -0.1774 | 0.8478 | -0.5637 | 0.8717 | -0.6790 | 0.2317 | 0.8282 | -0.5404 | 0.7373 | 0.7966 | -0.5579 | -0.5775 | 0.8648 | -0.6274 | 0.8785 | 0.7836 |
| Mn | 0.9050 | 0.0430 | 0.3984 | -0.4846 | 0.6792 | -0.7892 | 0.9346 | -0.8109 | 0.9777 | -0.4998 | -0.6312 | 0.5656 | -0.7500 | -0.3305 | 0.8753 | 0.9751 | -0.8464 | 0.9561 | -0.8479 | -0.6371 |
| Mo | 0.3450 | 0.5056 | 0.4081 | 0.1306 | 0.0076 | -0.6116 | 0.3644 | -0.5992 | 0.5542 | 0.2756 | -0.6084 | 0.6579 | -0.6619 | -0.5140 | 0.4616 | 0.5091 | -0.6364 | 0.4987 | -0.6353 | 0.6135 |
| Nb | -0.4592 | -0.3518 | -0.6327 | 0.1373 | -0.2038 | 0.9876 | -0.5442 | 0.9782 | -0.7498 | 0.0643 | 0.9935 | -0.6509 | 0.9734 | 0.8875 | -0.7503 | -0.6347 | 0.9595 | -0.7510 | 0.9229 | 0.5195 |
| Ni | 0.9173 | -0.0270 | 0.4174 | -0.5236 | 0.7065 | -0.8234 | 0.9577 | -0.8448 | 0.9982 | -0.5213 | -0.6662 | 0.5157 | -0.7777 | -0.3582 | 0.9132 | 0.9875 | -0.8751 | 0.9819 | -0.8720 | -0.7559 |
| P | 1.0000 | -0.2446 | 0.2250 | -0.6497 | 0.8568 | -0.5787 | 0.9545 | -0.6082 | 0.9098 | -0.6621 | -0.3792 | 0.3124 | -0.5247 | -0.0169 | 0.7869 | 0.9455 | -0.6365 | 0.9033 | -0.6581 | -0.6400 |
| Pb | -0.2446 | 1.0000 | 0.3137 | 0.5538 | -0.4779 | -0.2618 | -0.2162 | -0.2368 | -0.0187 | 0.4944 | -0.3812 | 0.8222 | -0.3441 | -0.4868 | 0.0221 | -0.0625 | -0.2396 | -0.0116 | -0.2338 | -0.2120 |
| Sb | 0.2250 | 0.3137 | 1.0000 | -0.0687 | 0.0477 | -0.6070 | 0.2860 | -0.6051 | 0.4362 | -0.0406 | -0.6118 | 0.4737 | -0.6116 | -0.5971 | 0.4438 | 0.3511 | -0.5993 | 0.4151 | -0.5877 | -0.6447 |
| Sn | -0.6497 | 0.5538 | -0.0687 | 1.0000 | -0.7195 | 0.2349 | -0.6546 | 0.2815 | -0.5212 | 0.8345 | 0.0716 | 0.1129 | 0.1054 | -0.1260 | -0.3867 | -0.5179 | 0.2820 | -0.4857 | 0.3066 | 0.6734 |
| Sr | 0.8568 | -0.4779 | 0.0477 | -0.7195 | 1.0000 | -0.3265 | 0.8135 | -0.3615 | 0.6950 | -0.7491 | -0.1337 | 0.0201 | -0.2579 | 0.2109 | 0.6732 | 0.7537 | -0.3759 | 0.7172 | -0.3860 | -0.8173 |
| Th | -0.5787 | -0.2618 | -0.6070 | 0.2349 | -0.3265 | 1.0000 | -0.6644 | 0.9971 | -0.8395 | 0.1750 | 0.9695 | -0.6311 | 0.9750 | 0.8162 | -0.8233 | -0.7410 | 0.9884 | -0.8356 | 0.9560 | 0.5764 |
| Ti | 0.9545 | -0.2162 | 0.2860 | -0.6546 | 0.8135 | -0.6644 | 1.0000 | -0.7018 | 0.9527 | -0.6695 | -0.4702 | 0.3501 | -0.5796 | -0.1420 | 0.8324 | 0.9769 | -0.7387 | 0.9287 | -0.7605 | 0.8445 |
| U | -0.6082 | -0.2368 | -0.6051 | 0.2815 | -0.3615 | 0.9971 | -0.7018 | 1.0000 | -0.8605 | 0.2226 | 0.9552 | -0.6256 | 0.9577 | 0.7957 | -0.8294 | -0.7673 | 0.9934 | -0.8527 | 0.9686 | 0.6511 |
| V | 0.9098 | -0.0187 | 0.4362 | -0.5212 | 0.6950 | -0.8395 | 0.9527 | -0.8605 | 1.0000 | -0.5072 | -0.6886 | 0.5194 | -0.7915 | -0.3858 | 0.9088 | 0.9832 | -0.8887 | 0.9794 | -0.8880 | -0.8109 |
| W | -0.6621 | 0.4944 | -0.0406 | 0.8345 | -0.7491 | 0.1750 | -0.6695 | 0.2226 | -0.5072 | 1.0000 | -0.0095 | 0.0553 | 0.0515 | -0.2271 | -0.3945 | -0.5408 | 0.2316 | -0.4929 | 0.2578 | 0.8152 |
| Y | -0.3792 | -0.3812 | -0.6118 | 0.0716 | -0.1337 | 0.9695 | -0.4702 | 0.9552 | -0.6886 | -0.0095 | 1.0000 | -0.6354 | 0.9626 | 0.9183 | -0.7061 | -0.5659 | 0.9330 | -0.6906 | 0.8911 | 0.4159 |
| Zn | 0.3124 | 0.8222 | 0.4737 | 0.1129 | 0.0201 | -0.6311 | 0.3501 | -0.6256 | 0.5194 | 0.0553 | -0.6354 | 1.0000 | -0.6687 | -0.5504 | 0.5031 | 0.4820 | -0.6448 | 0.5150 | -0.6415 | -0.7274 |
| Zr | -0.5247 | -0.3441 | -0.6116 | 0.1054 | -0.2579 | 0.9750 | -0.5796 | 0.9577 | -0.7915 | 0.0515 | 0.9626 | -0.6687 | 1.0000 | 0.8093 | -0.8215 | -0.6924 | 0.9520 | -0.7983 | 0.8989 | -0.5806 |
| Al_2_O_3_ | -0.0169 | -0.4868 | -0.5971 | -0.1260 | 0.2109 | 0.8162 | -0.1420 | 0.7957 | -0.3858 | -0.2271 | 0.9183 | -0.5504 | 0.8093 | 1.0000 | -0.4195 | -0.2334 | 0.7620 | -0.3759 | 0.7168 | 0.0112 |
| CaO | 0.7869 | 0.0221 | 0.4438 | -0.3867 | 0.6732 | -0.8233 | 0.8324 | -0.8294 | 0.9088 | -0.3945 | -0.7061 | 0.5031 | -0.8215 | -0.4195 | 1.0000 | 0.8871 | -0.8521 | 0.9386 | -0.8067 | -0.7263 |
| Fe_2_O_3_ | 0.9455 | -0.0625 | 0.3511 | -0.5179 | 0.7537 | -0.7410 | 0.9769 | -0.7673 | 0.9832 | -0.5408 | -0.5659 | 0.4820 | -0.6924 | -0.2334 | 0.8871 | 1.0000 | -0.8059 | 0.9672 | -0.8158 | -0.8300 |
| K_2_O | -0.6365 | -0.2396 | -0.5993 | 0.2820 | -0.3759 | 0.9884 | -0.7387 | 0.9934 | -0.8887 | 0.2316 | 0.9330 | -0.6448 | 0.9520 | 0.7620 | -0.8521 | -0.8059 | 1.0000 | -0.8697 | 0.9766 | 0.8122 |
| MgO | 0.9033 | -0.0116 | 0.4151 | -0.4857 | 0.7172 | -0.8356 | 0.9287 | -0.8527 | 0.9794 | -0.4929 | -0.6906 | 0.5150 | -0.7983 | -0.3759 | 0.9386 | 0.9672 | -0.8697 | 1.0000 | -0.8592 | -0.7960 |
| Na_2_O | -0.6581 | -0.2338 | -0.5877 | 0.3066 | -0.3860 | 0.9560 | -0.7605 | 0.9686 | -0.8880 | 0.2578 | 0.8911 | -0.6415 | 0.8989 | 0.7168 | -0.8067 | -0.8158 | 0.9766 | -0.8592 | 1.0000 | 0.7911 |
| SiO_2_ | -0.4190 | 0.4933 | 0.4262 | 0.3433 | -0.5696 | -0.4663 | -0.2959 | -0.4383 | -0.0669 | 0.4575 | -0.6450 | 0.3077 | -0.4655 | -0.8881 | -0.0281 | -0.2262 | -0.3880 | -0.0822 | -0.3532 | 1.0000 |
